# Barriers or gaps in implementation of misoprostol use for post-abortion care and post-partum hemorrhage prevention in developing countries: a systematic review

**DOI:** 10.1186/s12978-017-0383-5

**Published:** 2017-10-27

**Authors:** Amir Ali Barket Ali Samnani, Narjis Rizvi, Tazeen Saeed Ali, Farina Abrejo

**Affiliations:** Department of Community Health Sciences, The Aga Khan University, Stadium Road, Karachi, 74800 Pakistan

**Keywords:** Misoprostol, Post abortion care, Postpartum hemorrhage, Developing countries

## Abstract

**Background:**

Around 303,000 maternal deaths occur every year; most of these are preventable (World Health Organization), ICD-10: International classification of diseases and related health problems, 10th revision. Volume 2: Instruction manual, 2010). Ninety-nine percent of these maternal deaths occur in developing countries. PPH contributed 35 % (35%) of total maternal. Several interventions being done to reduce the number of maternal deaths. It has been noted that a simple low cost intervention of providing misoprostol timely could prevent these deaths.

**Objectives:**

The objectives of this systematic review was to identify barriers/gaps in the implementation of misoprostol use for prevention of postpartum hemorrhage and management of Post-abortion care services in developing countries.

**Methods:**

This study was a systematic review of published qualitative and quantitative literature on misoprostol in developing countries. Documents included were local and international peer reviewed articles and program reports on misoprostol implementation. PubMed, Google Scholars and Science direct databases were used along with Grey literature and manual search using terms “implementation gaps”, “misoprostol use”, “postpartum hemorrhage”, “post-abortion care” and “developing countries”.

**Results:**

Gaps or barriers in misoprostol use identified through systematic review can be categorized into six broader thematic areas including: inconsistency in supplies and its distribution; inadequate staffing; lack of knowledge of providers and end users, absence of the registration of drug and fear and apprehensions related to its use at provider and policy level.

**Conclusion:**

It is concluded that barriers and gaps can be addressed through providing enabling environment through supportive policies, designing a formal plan for supplies, task shifting strategies and use of guidelines and protocols for successful implementation.

**Electronic supplementary material:**

The online version of this article (10.1186/s12978-017-0383-5) contains supplementary material, which is available to authorized users.

## Plain English summary

Maternal death in developing countries is common across the globe. Post-partum hemorrhage (PPH) is the major contributor of these deaths. Oxytocin is considered as the first line drug and it is widely used by health care providers since decades to prevent and manage PPH but on the basis of emerging body of evidences World Health Organization (WHO) recommended misoprostol use for similar conditions in settings where oxytocin is not available and included misoprostol in its essential medicines list (EML) model in March 2011. Realizing the importance of misoprostol in averting postpartum hemorrhage and abortion related complications, misoprostol has been recommended for primary prevention of PPH.

However, many developing countries are facing barriers in proper implementation of misoprostol. This systematic review was aimed to identify barriers or gaps in the implementation of misoprostol use for prevention of postpartum hemorrhage and management of Post-abortion care services in developing countries; and suggest recommendations for its elimination.

Systematic review of published qualitative and quantitative literature on misoprostol in developing countries were search based on eligibility criteria using data bases that include; PubMed, Google Scholars and Science direct along with Grey literature and manual search using search terms “implementation gaps”, “misoprostol use”, “postpartum hemorrhage”, “post-abortion care” and “developing countries”. Total 19 studies were included for analyses purpose.

Based on review findings it is concluded that barriers and gaps can be addressed through providing enabling environment through supportive policies, designing a formal plan for supplies, task shifting strategies and use of guidelines and protocols for successful implementation (Additional file 1; Table S1).

## Background

Around 303,000 maternal deaths occur every year; most of these are preventable [1]. Ninety-nine percent of these maternal deaths occur in developing countries [2, 3]; with Sub-Saharan African and south Asian countries accounts for 66% (201,000) and 21.7% (187,000) respectively of total maternal mortality [1].

The overall Maternal Mortality Ratio (MMR) in developing countries is 239 per 100,000 live births which is 20 times higher than developed regions [1, 3]. Sub-Saharan African countries have very high MMR of 546/100,000 live births; whereas South Asian Countries have MMR of 176/100,000 live births. In Pakistan Maternal Mortality Ratio (MMR) is 178/100,000 live births which is still higher than MDGs targets of maternal mortality reduction [1].

Five direct causes of maternal mortality are; Postpartum hemorrhage (PPH), unsafe abortions (or related complications), eclampsia, obstructed labor and sepsis [4]; PPH contribute 35 % of total maternal deaths in developing countries and continue to be the leading cause of maternal mortality [1]. PPH is the predominate cause of maternal mortality in Africa (34%) and Asia (31%) [5] where most of the maternal deaths occur. Despite under-reporting because of stigma [6] abortion still is a major contributor of maternal deaths; in 2013; approximately 15% maternal deaths occurred globally as a result of abortion related complications [7].

Oxytocin is considered as the first line drug and it is widely used by health care providers since decades to prevent and manage PPH but on the basis of emerging body of evidences World Health Organization (WHO) recommended misoprostol use for similar conditions in settings where oxytocin is not available and include misoprostol in its essential medicines list (EML) model in March 2011 [5]. As a result, WHO 2012 guidelines for PPH management recommend the administration of misoprostol by Community Health Workers (CHWs) for PPH prevention [5]. Misoprostol has been 95% effective for managing incomplete abortion in poor resource settings [8]. Moreover, the drug is the best acceptable and effective substitute of Manual Vacuum Aspiration (MVA) for incomplete abortion [9]. Realizing the importance of misoprostol in averting postpartum hemorrhage and abortion related complications, misoprostol has been recommended for primary prevention of PPH [10].

Despite these recommendations; developing countries have yet not successfully implemented misoprostol use to prevent PPH and reduce maternal deaths. This therefore has identified the need to explore barriers or gaps hindering misoprostol implementation [9].

Pakistan is among low-resource setting countries where misoprostol could be the drug of preference to reduce deaths from PPH as majority of the deliveries take place at home assisted by Dai (a local term used for TBAs) or family member [11].

**Fig. 1:**
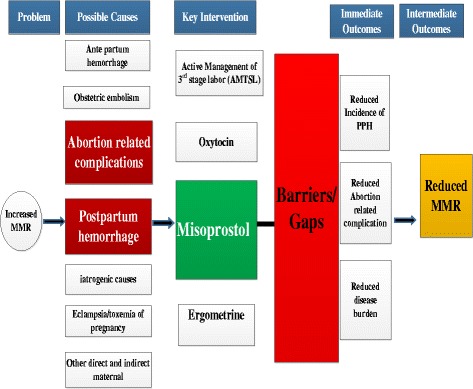
Conceptual framework

The objectives of this systematic review were to: identify barriers or gaps in the implementation of misoprostol use for prevention of postpartum hemorrhage and management of Post-abortion care services in developing countries; and suggest recommendations for elimination of barriers or gaps in implementation of misoprostol to reduce maternal mortality in developing countries. The below Fig. 1 depicts the conceptual framework designed by the author specific to the context of this study to understand the overall approach of this study (Additional file 1; Table S2).

## Methods

This study was a systematic review of published Qualitative and Quantitative literature from March, 2012 to July, 2016 on implementation of misoprostol in developing countries. Preferred reporting items for systematic review and Meta-Analysis (PRISMA) checklist has been used for this systematic review (Additional file 2). PRIMSA comprised of 27 items checklist (refer Additional file 1; Table S3 below). The aim of PRISMA statement is to assist author to improve systematic review reporting [12].

Search strategy was developed to identify publications and project reports that had explored barriers/ gaps in implementation of misoprostol using three data bases concomitantly; that include, Google Scholars, PubMed, and Science direct using search strategy, “Implementation gaps OR barriers AND misoprostol use AND Postpartum hemorrhage AND post abortion care AND developing countries”.

**Fig. 2:**
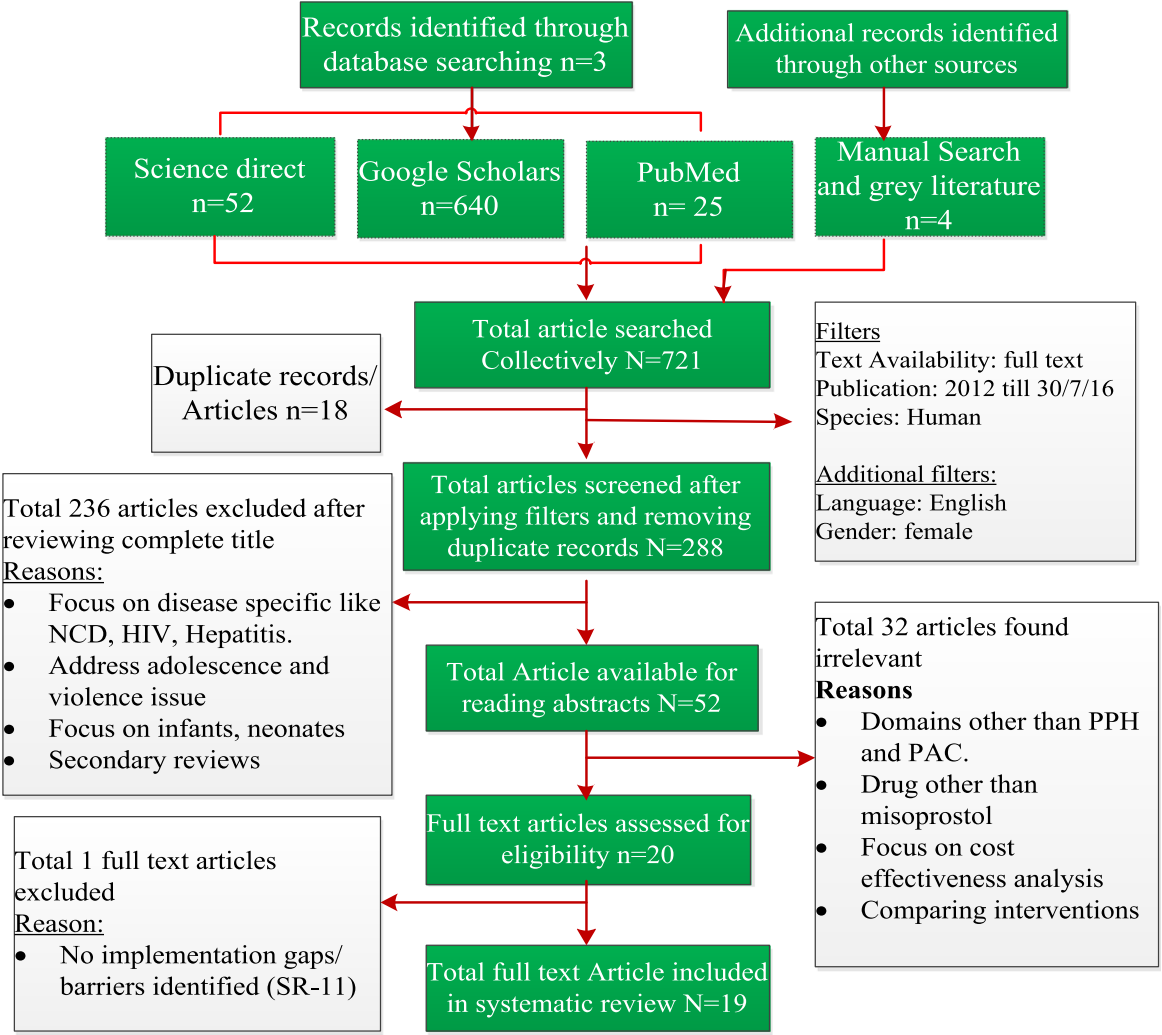
Consort diagram for data screening process
- Intervention: Misoprostol for prevention Post-Partum Hemorrhage (PPH) and for management of Post abortion care (PAC)
- Comparison: Since this review is not the clinical trial, rather a health system research hence there is no comparison group
- Outcome: the considered outcome could be any one of the following 1) reduced incidence of PPH 2) reduced abortion related complications 3) reduced maternal morbidity4) reduced maternal mortality. Since outcome assessment is beyond the scope of this systematic review, therefore the possible outcomes were not assessed.

Following are the eligibility criteria for including or excluding articles included; Year of publication: March, 2012 till July, 2016 (because in March, 2012; the WHO guidelines for the prevention and management of PPH have included a recommendation for the administration of misoprostol by CHWs for the prevention of PPH) [5]. Focus should be on Human species, Published articles must be in English language, Gender: female (female related conditions), Developing country context, Drug of focus: misoprostol, Maternal condition related to Postpartum Hemorrhage (PPH) prevention, Post Abortion care (PAC) services, Articles/reports (grey literature) included irrespective of the publication status if qualifying the selection criteria (refer Fig. 2, below).

- Population: Developing countries where the misoprostol intervention is implemented.

A self-designed data extraction form was developed through an iterative process to recognize all data set that considered most critical to this review objective. Information gathered from eligible studies comprised of name of journal, date of review, publication year, study settings, study design, methodology for data collection, targeted audience, variable of interest, implementation barriers or gaps identified, advantages of misoprostol, either excluded or not, conclusion and recommendations/ policy implications. Lastly, the information extracted from 20 included studies were documented in extraction form (Additional file 1; annexure 1.1-1.20).

### Methodological assessment of quality of this systematic review

An AMSTAR checklist was used as a measurement tool to assess the methodological quality of this systematic review. Principles of AMSTAR tool can be used to demonstrate aspects of systematic review methodology that influence the overall quality of review. An overall score relating to review quality was calculated. AMSTAR Characterized quality at three level includes high quality (Score ranges from 8 to 11), medium quality (score ranges from 4 to 7) and low quality (score ranges from 0 to 3) [13]. The methodological assessment of this systematic review revealed total score of 9 which reflects high quality of systematic review (Additional file 1; Table S4).

### Quality assessment of included studies

The Mirza and Jenkins checklist was used for assessing the quality of each included studies. The quality assessment criteria include 1) Explicit study aims stated 2) justification of sample size given 3) sample representation of population 4) inclusion and exclusion criteria stated 5) reliability and validity of measured justified 6) response rate and dropout rate specified 7) data adequately described 8) statistical significance assessed 9) discussion on generalizability given 10) null finding interpreted [14]. Out of 19 included studies, only single study gave complete methodological details as per checklist.

Based on Quality of evidence criteria: score ≤ 5 is low; score of >5 and ≤7.5 is moderate; and score of ≥7.6 is high. Out of 19 studies, the 13 studies (*n* = 13) were categorized as moderate; whereas six studies (*n* = 6) were categorized has high quality studies. However only single study falls under low quality evidence thus excluded from this systematic review (Additional file 1; Table S5).

## Results

As mentioned above, from the list of 20 shortlisted articles for full text review, one study was excluded as it was not capturing any barrier or gap related to misoprostol. The selected 20 studies were run from pre-formulated data extraction form that includes; study citation, objectives, study settings, study design and data collection methods, barriers identified, conclusion, recommendations and limitations of the study.

Below Table 1 depicts the summarize findings contained study settings and study design from selected studies.

**Table 1:** Summary of findings

Study Settings	Developing countries that include, Bangladesh, Afghanistan, Ethiopia, Ghana, Kenya, Uganda, Northern Nigeria, Tanzania, India, Pakistan Kosovo, Malawi, Myanmar, Sub-Saharan African countries (Democratic Republic of Congo, Maban & Burkina Faso).
Study design	• Qualitative inquiry using In-depth interviews and FGD’s (*n* = 7) • Mixed method design (n = 3) • Situational analysis (*n* = 3), • Cross-sectional study design (n = 2) • Before and after study design (*n* = 2). • Web based survey (n = 1) • Evaluation approach as study design (*n* = 1). • Special communication (n = 1)

All the studies included in this review were from developing countries. The findings from the included studies were categorized into six categories. These six categories were further divided into sub-categories and each finding is segregated accordingly (refer Table 2 below).

**Table 2:** Barriers or gaps identified from included articles

S.No	Health System Building Blocks/Thematic Areas	Sub-Themes for identified gaps or barriers	Number of studies identified similar gap/barriers
1	Barriers or gaps related to Leadership, governance, Guidelines and Policy related (12 Studies)	Lack of national policies and guidelines for MPAC or PPH	2
		No Registration of misoprostol Specific for PAC or PPH	3
		Fear and apprehensions related to its use	3
		Misoprostol not included in National list of essential medicines	1
		legal restrictions that only permits pregnancy termination to save life of mother	1
		No or Less Preference to misoprostol	1
		Lack of integration of misoprostol with Basic package of health services/ health care resources	2
		Poorly developed Commodity security strategies.	1
		Rudimentary or absent Transportation and communication system.	2
		Lack of ability to implement and monitor implementation and current practices.	1
		Lack of trust between clinicians and policy makers	1
		Gaps in pre-service medical and midwifery education program curriculum.	1
		Technical inconsistencies and ambiguity in guidelines and protocols- incomplete and out dated.	2
		Lack of communication or awareness of existing policy	2
2	Barriers or gaps related to Health Service Delivery, and availability and access to essential medicine (10 Studies)	Lack of Access to misoprostol	2
		PAC services not available	1
		Issues related to inconsistencies in supplies/ fragmented supply chain and distribution	8
		Lack of Supervision/Monitoring capacity	1
		Reduced institutional delivery	1
		Lack of provider preference of Medical Abortion using misoprostol	1
3	Barriers or gaps related to Health Workforce (14 studies)	Lack of Knowledge & Skills (Technical & non –technical) of providers	3
		Lack of training and training capacity of providers	2
		scarcity of staff or inadequate staffing (SBA’s, TBA’s, CHW’s)	6
		Fear, apprehensions and doubt related to misoprostol	5
		Negative or Judgmental attitude of providers	1
		Limited scope of practice of midwifes	1
		Lack of awareness/Clarity of the guidelines/evidence.	4
		Lack of communication/inter-professional collaboration	2
		Lack of Motivation among provider	1
4.	Issues related to Community perception, Knowledge and preference: (8 Studies)	Lack of acceptability and negative attitude due to stigma associated due to its abortion inducing properties	3
		Hindrance from relatives in taking misoprostol	1
		Lack of community awareness and knowledge for misoprostol	2
		Lack of health seeking behavior	2
		Lack of preference to Medical Abortion using misoprostol	1
		Lack of access to misoprostol due socio-economic, Ethnic and cultural barriers	2
		Patients’ lack of trust of lower-cadre health workers	1
		Disparities in service utilization between rural and urban	1
5	Barriers or gaps related to Health information system (1 Study)	Lack of national reporting on HMIS on use of uterotonics.	1
		Gaps in inclusion of maternal health indicators in national data	1
6	Barriers or gaps related to cost of medicine (2 Studies)	Paying for medicine is a bottle neck to improve coverage despite to be inexpensive	2
		Financial constraints in term of training TBA’s, cost of drug	1

The thematic areas reflected the health system building block framework proposed by WHO in 2007 describing health system in term of six core components that include Health service delivery, Health workforce, Health information systems, access to essential medicines, health care financing and health care leadership/governance [15, 16]. In context to this systematic review author has slightly modified these health system building blocks framework by combining the two building blocks that are health service delivery and access to essential medicine and adding issues related to community knowledge and perception; since many studies have highlighted barriers pertaining to this particular thematic area.

1. Barriers or gaps related to Leadership, governance, Guidelines and Policy related
  The majority of studies (12/19) had identified that leadership, governance and policy related issues are substantial barriers in successful implementation of misoprostol in developing countries. More specifically, lack of registration of misoprostol for the management of PAC or PPH were highlighted (*n* = 3) [9, 11, 17].
  Few studies (n = 3) had identified existence of fear and confusion among implementers, policy makers and government officials [17–19]. Specifically lack of awareness about existing policy (*n* = 2) [9, 20] and lack of integration of misoprostol in basic health service Package (n = 2) were important barriers found [21, 22], moreover it was also explored that there were technical inconsistencies and ambiguity in guidelines and protocols at policy level [17, 23].
  Under the similar domain two studies have identified Rudimentary or absent transportation (related to road infrastructure) and communication system as barrier [18, 24].
  A Cross-sectional study from 37 developing countries using Key informant interviews revealed that majority of the countries have included Oxytocin in essential medicine list and have less preference for misoprostol to prevent PPH [23] also misoprostol use to prevent PPH at home birth have piloted in some countries but far fewer have taken the strategy to scale up [23].
2. Barriers or gaps related to Health service delivery, and availability and access to essential medicines:
  More than half of the studies (*n* = 10/19) identified barriers or gaps related to health service delivery and access to essential medicine. Among these the most frequently reported was issues related to inconsistencies in supplies/ fragmented supply chain and distribution (*n* = 8/10) studies, have reported this similar issue [7, 9, 18, 19, 22–25]. Remaining two studies (*n* = 2) out of 19 have identified lack of access to misoprostol as the barrier for increasing coverage and optimal utilization [19, 24].
3. Barriers or gaps related to Health Workforce:
  Significant number of studies (14/19) have identified gaps or barriers related to health workforce. The most repeatedly reported gap/barrier were scarcity or inadequate staffing of SBA’s, TBA’s, and CHW’s (*n* = 6) [9, 18, 21, 24–26] fear and confusion of the providers (*n* = 5) [8, 17, 22, 25, 27]. Fear in term of misuse of drug due to its abortion inducing properties or administration in event of an undiagnosed twin (lead to fatal outcome), administration during labor (causing uterine rupture) and use for unsafe abortion [17], furthermore same study also mentioned that in response to advance distribution; fears concerning intra-partum administration, undiagnosed multiple gestation, medication sharing and possibility of its use after expiry were also the added fear that could jeopardize misoprostol distribution. In addition to this it was also mentioned in same study that, it is believed by some the providers that misoprostol will increase the home based delivery and limit the facility based delivery and thus deviating away from the strategy to increase use of facilities for birth [19]. Lack of awareness of the existence of guidelines and clarity of guidelines (*n* = 4) [11, 20, 22, 24] and lack of knowledge and Skills of providers was also highlighted (*n* = 3) [7, 25, 28].
4. Barriers related to Community perception, Knowledge and preference:
  Nearly half of the studies (8/19) have identified barriers or gaps related to community perception, knowledge and preference. Most common factors in this domain were; lack of acceptability and negative attitude due to its abortion inducing properties (n = 3) [7, 11, 26]; lack of access to misoprostol due socio-economic, Ethnic and cultural barriers (*n* = 2) [22, 27]; lack of community awareness and knowledge for misoprostol (n = 2) [26, 29]; and inequitable distribution of misoprostol is an equitable intervention that can reduce disparities in access to it [21].
5. Barriers or gaps related to Health Information System:
  Only single study out of 19 studies has identified barriers or gaps related to health management information system that include absence of national reporting system on uterotonics use, along with gaps in inclusion of key maternal health indicators at national level impedes and continues to limit progress. Combining enough data on implementation of critical interventions is crucial to warrant that these interventions are prioritized and that progress is measures [5].
6. Issues related to cost of medicine:
  Only two studies (2/19)) have identified issues related to the cost of medicine. A study conducted in 37 developing countries focused on national level findings revealed that Paying for medicine is a bottle neck to improve coverage [5]. Similar findings were shared by report on situational analysis conducted in Ethiopia in 2012; and it was found that cost of medicine was an issue [24]. Similar study further added that there were financial constraints also in term of training TBA’s [24].

## Discussion

PPH is major contributor of maternal mortality in developing countries and accounts for more than 30% of maternal mortality in African and South Asian countries [5]. Treating or preventing PPH with misoprostol can avoid many complications and can reduce these preventable deaths. Large scale implementation of misoprostol would contribute in achieving 2030- agenda of global commitment that is to reduce Average global maternal mortality ratio (MMR) of less than 70 maternal deaths per 100,000 live births by 2030 along with Supplementary national target that by 2030, no country should have an MMR greater than 140, a number twice the global target [1, 3].

This review exhibited variations in misoprostol implementation from one geographic territory to another for prevention of PPH and abortion related complications. Literature regarding misoprostol implementation programs from developing countries illustrates that barriers or gaps exist at all three levels of health system; community, facility and policy/ national level.

The most important barrier or gap for using Misoprostol to prevent PPH was inconsistencies in supplies/ fragmented supply chain and distribution of misoprostol resulting into frequent stock out. A study conducted in Kenya argued that institutional failure to allocate budget to procure misoprostol was also resulted in delayed acquisition of this drug by 1 month [9]. A strategy to make misoprostol widely available at community level specific for prevention of post-partum hemorrhage also faced political hurdles over its perceived misuse was the another possible reason for the inconsistencies in supply chain [19].

Consistent with above discussed reasons, policy or system related causes are also linked with this frequently highlighted supply and distribution issue. Review findings explored that some program officials and policy makers were might be reluctant to promote a community-based maternal health intervention due to existence of fear and apprehensions related to its use and promotion [17–19]; they feel that home based distribution of misoprostol could increase the home based deliveries or it could be misused due to its abortion inducing properties, and possibly will divert attention away from implementing oxytocin-- a superior drug. An ambiguity, fear and apprehensions among key stake holders on aforementioned distresses were identified as potential cause for underlying barrier.

Among other barriers at policy level includes lack of clear guidelines and unavailability of misoprostol labelled for PPH specifically further increase the confusion. There was also a fear of empowering women to participate in their health care and the controversy surrounding the use of misoprostol for abortion. In countries where abortion is still restricted by law, there is a possibility of greater conflict if misoprostol is registered specifically for PAC or for advance community based distribution, perhaps this would be abused for illegal abortion as it happened in Latin America, that burst out into intense political controversy. The findings from multiple studies also witnessed perceived misused and abortion inducing properties of misoprostol as the reason for non-registration of misoprostol specific for PPH and for advance distribution of misoprostol in several developing countries.

In addition to this; there were risk associated with limited community knowledge regarding dosage and timings and provider knowledge to differentiate between PPH caused by atony or due to other causes such as uterine rupture, vaginal lacerations and placental abnormalities; that have also increased the fear and confusion at policy level to registered misoprostol in some of the developing countries. One of the study has opposing view in this regard and mentioned that not registering mean that a marketing agreement is not formally in place to permit its promotion and sale of drug for particular indication, it was further added that government are not beholden to commercially registered and choose to make a product available if it has public benefits [17]. Pursuing registration of misoprostol may in some cases be useful to strategize for ensuring increase availability.

It has been established from the above discussion that there was common root cause behind both the main issues that was fear and apprehensions among stake holders (that includes policy makers, government officials and providers) that had a serious consequence in supply and distribution of misoprostol and on registration status of misoprostol (as in Kenya). However, literature has identified fear and apprehension explicitly as a separate barrier at provider level. The findings from Ethiopia witnessed this fact that staff experience fear or safety concerns includes giving misoprostol in a condition of undiagnosed twin (leading to fatal outcome), during labor (causes uterine rupture), and use of unsafe abortion. In response to advance distribution; fear concerning intra-partum administration, undiagnosed multiple gestation, unsafe abortion, medication sharing and possibility of its use after expiries were the major concern raised.

Beyond ensuring consistent supplies and uninterrupted distribution of misoprostol; another major contributor revealed from this review that impede proper implementation of misoprostol was scarcity of staff or inadequate staffing (SBA’s, TBA’s, CHW’s), staff requirement for the provision of PAC services were minimal. This staffing shortage was also found at community level, because access to SBA’s in remote areas was quite difficult in developing countries. Misoprostol distribution through CHWs’ achieved high coverage [22] but it is highly uncertain that to what extent does CHW’s reach women resides in remote rural areas. Many conventional programs exclusively emphasized on increasing access to SBA’s with the use of TBA’s in the role to advocate for skills care only. TBA’s were utilized in a role that primarily urged women to obtain antenatal, obstetrics and postnatal care with little attention of enhancing the skills set and knowledge base of TBA’s [30].

The programs that facilitates community based distribution of misoprostol represents such an opportunity to think and implement task shifting strategies. This highlighted the need for a comprehensive approach rather than focusing on single agenda of distribution coverage. A success story of Northern Nigeria to improve supplies and access to misoprostol by enhancing the community based distribution of misoprostol by introducing the new cadre of community based drug keepers and trained them; is feasible, safe and acceptable intervention in settings where home based deliveries were high and uterotonic coverage is limited. It not only provides protection to women delivering at home but also helped in overcoming structural and cultural barriers that limits women access to health facilities [28].

Further to above discussed issue, incomplete and inconsistent knowledge of provider about misoprostol including appropriate dosage, timings, adverse effects, and required monitoring. Possible reasons explored from studies include; training did not follow current guidelines, also existing trainings are more inclined towards general management rather than current PAC related guidelines, Gaps in pre-service medical and midwifery education program curriculum, unavailability of refresher training to determine the ability to retained provided knowledge and learnt skills. This inconsistency of provider knowledge and limited skilled set has implications for further training programs. Training additional number of CHWs/TBA’s as an alternative strategy to reach women without access to existing health services. Incorporating pre-service training using updated curriculum in light of current guidelines would facilitate in the resolving above discussed staffing and capacity issue.

Despite some concerns about stigma associated due to its abortion inducing properties and perceived misuse in above discussion among both audiences (providers and end users), however review did not explore sufficient evidences related to acceptability barriers, lack of trust in lower-cadre health workers, barriers due to ethnic and cultural differences, disparities in service utilization between rural and urban.

### Strengths of study

The main Strengths of this systematic review are the context specific problem that have evaluative policy implications. Secondly systematic review made use of both qualitative and quantitative set of data to better understand the barriers and their underlying reasons.

### Study limitations

Data limitations, since it was a secondary analysis.

### Conclusion and recommendations

## Conclusion

Although literature demonstrates significant role Misoprostol use can play in prevention and management of PPH and safe PAC services in context to developing countries, yet the impact of this intervention on maternal indicators can be visible only once the intervention is implemented and sustained. This requires bridging of identified gaps and overcoming barriers by developing supportive policies that actually overcome the fear and apprehensions at policy, provider and community level, training of health care providers including CHW’s and TBA’s for its appropriate use and counsel them to overcome fears and apprehension/myths associated with its use, correctly estimating the demand and designing a formal plan to maintain adequate supplies and to prevent frequent stock out of misoprostol. It would also require task shifting strategies to overcome inadequate or shortage of staffing.

These findings might be informative for the government officials, NGO’s, program personnel and policy makers to implement efficiently, effectively and scale up for sustainability to save women’s life.

### Recommendations and policy implications

Findings of this systematic review have public health implications for programs in context to its availability, feasibility, sustainability and scaling-up in better implementing misoprostol in developing countries. Below mentioned are the some of the recommendation proposed in light identified findings

- *Policy level:*
  - Countries must establish supportive policies for PPH prevention, PAC services and advance distribution of misoprostol in line with international standards that reflect the latest research and the most recent WHO recommendations.
  - Registering misoprostol for PPH, PAC services and for the management of incomplete abortion and correctly approximating demand of misoprostol.
  - Use of mass media campaigns, education materials and community champions for extensive dissemination of guidelines beyond distributing them directly to users.
  - Health monitoring and reporting system need to be improved by tracking maternal health related indicators, also PAC related service information should be integrated into National HMIS and national monitoring checklist
- *Facility/ provider Level*
  - Designing a formal plan to maintain adequate supplies and to prevent frequent stock out of misoprostol.
  - Regular supervision and supportive mentoring to ensure compliance to guidelines and protocols.
  - TBA’s would be utilized in a role that primarily urged women to obtain antenatal, obstetric and post-natal care. This task shifting strategies will continue to encounter barriers related to limited or inadequate staffing.
  - Provide training/ orient health care providers including CHW’s and TBA’s on policies and guidelines for its appropriate use and to make them aware of existing policies and to overcome/clarify fear and apprehensions/myths associated with its use.
- *Community Level:*
  - Strengthen awareness raising efforts with communities and families to communicate the potential benefits, correct usage of misoprostol, counselling on danger signs, importance of increasing facility births and clarification of myths and misconceptions in order to effectively utilize it and overcoming the community related concerns.
  - Developing education and behavior change communication (BCC) materials for all relevant cadres and community members to ensure its appropriate use, dosage, adverse effects and reactions.

## Additional files

Additional file 1: Annexes or list of tables. (DOCX 172 kb)

Additional file 2: PRISMA Checklist. (DOC 52 kb)

Additional file 3: ERC Approval / exemption letter. (DOCX 771 kb)

## Abbreviations

AKU: Aga Khan University
AMTSL: Active management of third stage labor
ANC: Ante-natal care
BCC: Behavioral change communication
CHS: Community Health Sciences
CMWs: Community Health workers
DRC: Democratic Republic of Congo
EML: Essential Medicine list
ERC: Ethical Review Committee
FIGO: International Federation of Gynecology and Obstetrics
FP: Family planning
HCPs: Health care providers
HMIS: Health management information system
MA: Medical Abortion
MoH: Ministry of health
MPAC: Misoprostol for Post abortion care
MVA: Manual vacuum aspiration
OOPP: Out of pocket payment
PAC: Post Abortion care
PPH: Postpartum hemorrhage
RCT: Randomized control trial
SBAs: skilled birth attendants
SR: Systematic Review
WHO: World Health Organization
