# Supplementary material for: Barriers or gaps in implementation of misoprostol use for post-abortion care and post-partum hemorrhage prevention in developing countries: a systematic review
Source: Reprod Health. 2017 Oct 27;14:139. doi: 10.1186/s12978-017-0383-5 (PMC5659002; doi:10.1186/s12978-017-0383-5)
Supplement: Supplementary file 1 — Annexes or list of tables. (DOCX 172 kb) [file 12978_2017_383_MOESM1_ESM.docx]

**“IMPLEMENTATION BARRIERS OR GAPS IN MISOPROSTOL USE FOR POST-ABORTION CARE AND POST-PARTUM HEMORRHAGE PREVENTION IN DEVELOPING COUNTRIES: A SYSTEMATIC REVIEW”**

Author

**DR. AMIR ALI S/0 BARKET ALI SAMNANI**

Co-Author

**DR. NARJIS RIZVI**

**DR. TAZEEN SAEED ALI**

**Ms. FARINA ABREJO**

Karachi, Pakistan

October, 2016

**Tables and figures**

Contents

[**List of Tables** 3](#_Toc479430797)

[**Table S1 Definition of Key words:** 3](#_Toc479430798)

[**Table S2: Findings from Literature review** 4](#_Toc479430799)

[**Table S3: Summary of findings** 5](#_Toc479430800)

[**Table S4: Barriers or gaps identified from included articles** 6](#_Toc479430801)

[**Table S5: Quality Assessment of included studies:** 9](#_Toc479430802)

[**Table S6: Barriers or gaps identified from included articles** 11](#_Toc479430803)

[**Table S7: PRISMA Checklist** 12](#_Toc479430804)

[**Table S8: Filled data Extraction Forms:** 14](#_Toc479430805)

[**Table S8.1** 14](#_Toc479430806)

[**Table S8.2** 16](#_Toc479430807)

[**Table S8.3** 18](#_Toc479430808)

[**Table S8.4** 20](#_Toc479430809)

[**Table S8.5** 22](#_Toc479430810)

[**Table S8.6** 24](#_Toc479430811)

[**Table S8.7** 25](#_Toc479430812)

[**Table: S8.8** 27](#_Toc479430813)

[**Table: S8.9** 28](#_Toc479430814)

[**Table: S8.10** 30](#_Toc479430815)

[**Table S8:11** 32](#_Toc479430816)

[**Table: S8.12** 33](#_Toc479430817)

[**Table S8.13** 35](#_Toc479430818)

[**Table S8.14** 37](#_Toc479430819)

[**Table S8.15** 39](#_Toc479430820)

[**Table S8.16** 41](#_Toc479430821)

[**Table S8.17** 43](#_Toc479430822)

[**Table S8.18** 45](#_Toc479430823)

[**Table S8.19** 47](#_Toc479430824)

[**Table S8.20** 49](#_Toc479430825)

[**Table S9:**  **List of Excluded and Included Studies:** 51](#_Toc479430826)

[**Table S10: Methodological details and key findings of the included studies:** 54](#_Toc479430827)

[**Table S11: AMSTAR- a measurement tool to assess the methodological quality of**   **Systematic Review** 59](#_Toc479430828)

**List of Tables**

## **Table S1 Definition of Key words:**

| **Key words** | **Definition** |
| --- | --- |
| Misoprostol | The American Society of Health-System Pharmacists defined misoprostol as prostaglandin –E1 analogue, a medication used to start labor, cause an abortion, prevent and treat stomach ulcers, and treat postpartum bleeding due to poor contraction of the uterus. |
| Postpartum Hemorrhage | World health organization has defined PPH as “blood loss of 500 ml or more within first 24 hours of childbirth” |
| Post Abortion care | Post abortion care is an integral component of comprehensive abortion care and includes five essential elements: Treatment of incomplete and unsafe abortion and complications. Counseling to identify and respond to women's emotional and physical health needs (IPAS) |
| Developing country | According to Wikipedia developing country is a nation with a less developed industrial base, and a low Human Development Index (HDI) relative to other countries |

## **Table S2: Findings from Literature review**

| **Kenya**  Osur, J., Baird, T. L., Levandowski, B. A., Jackson, E., & Murokora, D. (2013). | 1. Lack of national policies and guidelines for MPAC. 2. No Registration of misoprostol specifically for PAC |
| --- | --- |
| **India**  Deepak, N. N., Mirzabagi, E., Koski, A., & Tripathi, V. (2013). | 1. Inconsistency of provider knowledge related to its dosage, required monitoring, and its administration |
| **Pakistan**  Mir, A. M., Wajid, A., & Gull, S. (2012). | 1. Hindrance from relatives in taking misoprostol (opposing view) 2. Lack of knowledge of study participants regarding timings of taking misoprostol and appropriate timings for referrals |
| **Bangladesh**  Prata, N., Bell, S., Holston, M., & Quaiyum, M. A. (2014). | 1. Inadequate Coverage of SBAs and TBA’s |
| **Ghana**  Aniteye, P., & Mayhew, S. H. (2013). | 1. Perceived lack of administrative support. 2. Judgmental attitudes because of personal, social and structural reasons |
| **Afghanistan**  Haver, J., Ansari, N., Zainullah, P., Kim, Y. M., & Tappis, H. (2016). | 1. Lack of integration of advance distribution of misoprostol with Basic package of health services |

## **Table S3: Summary of findings**

| **Study Settings** | Developing countries that include, Bangladesh, Afghanistan, Ethiopia, Ghana, Kenya, Uganda, Northern Nigeria, Tanzania, India, Pakistan Kosovo, Malawi, Myanmar, Sub-Saharan African countries (Democratic Republic of Congo, Maban & Burkina Faso). | |
| --- | --- | --- |
| **Study design** | - Qualitative inquiry using In-depth interviews and FGD’s (n=7) - Mixed method design (n=3) - Situational analysis (n=3), - Cross-sectional study design (n=2) - Before and after study design (n=2). - Web based survey (n=1) - Evaluation approach as study design (n=1). - Special communication (n=1) | |
| **Outcome (measures to reduce maternal mortality)** | **Number of studies** | **Quality of evidence**  **(Average Quality Score)** |
| Reduction in incidence of postpartum hemorrhage | 14 Studies | Moderate quality  (Quality score 7.07) |
| Reduction in abortion related complications | 3 Studies | High Quality  (Quality score 8.33) |
| Reduction in disease burden (morbidity) | 2 studies | Moderate Quality  (Quality Score 6.5) |

*Quality of evidence criteria: score ≤ 5 is low; score of >5 and ≤ 7.5 is moderate; and score of ≥ 7.6 is high

## **Table S4: Barriers or gaps identified from included articles**

| **S.No** | **Health System Building Blocks/Thematic Areas** | **Sub-Themes for identified gaps or barriers** | **Number of studies identified similar gap/barriers** |
| --- | --- | --- | --- |
| 1 | Barriers or gaps related to Leadership, governance, Guidelines and Policy related  (12 Studies) | Lack of national policies and guidelines for MPAC or PPH | 2 |
|  |  | No Registration of misoprostol Specific for PAC or PPH | 3 |
|  |  | Fear and apprehensions related to its use | 3 |
|  |  | Misoprostol not included in National list of essential medicines | 1 |
|  |  | legal restrictions that only permits pregnancy termination to save life of mother | 1 |
|  |  | No or Less Preference to misoprostol | 1 |
|  |  | Lack of integration of misoprostol with Basic package of health services/ health care resources | 2 |
|  |  | Poorly developed Commodity security strategies. | 1 |
|  |  | Rudimentary or absent Transportation and communication system. | 2 |
|  |  | Lack of ability to implement and monitor implementation and current practices. | 1 |
|  |  | Lack of trust between clinicians and policy makers | 1 |
|  |  | Gaps in pre-service medical and midwifery education program curriculum. | 1 |
|  |  | Technical inconsistencies and ambiguity in guidelines and protocols- incomplete and out dated. | 2 |
|  |  | Lack of communication or awareness of existing policy | 2 |
| 2 | Barriers or gaps related to Health Service Delivery, and availability and access to essential medicine  (10 Studies) | Lack of Access to misoprostol | 2 |
|  |  | PAC services not available | 1 |
|  |  | Issues related to inconsistencies in supplies/ fragmented supply chain and distribution | 8 |
|  |  | Lack of Supervision/Monitoring capacity | 1 |
|  |  | Reduced institutional delivery | 1 |
|  |  | Lack of provider preference of Medical Abortion using misoprostol | 1 |
| 3 | Barriers or gaps related to Health Workforce  (14 studies) | Lack of Knowledge & Skills (Technical & non –technical) of providers | 3 |
|  |  | Lack of training and training capacity of providers | 2 |
|  |  | scarcity of staff or inadequate staffing (SBA’s, TBA’s, CHW’s) | 6 |
|  |  | Fear, apprehensions and doubt related to misoprostol | 5 |
|  |  | Negative or Judgmental attitude of providers | 1 |
|  |  | Limited scope of practice of midwifes | 1 |
|  |  | Lack of awareness/Clarity of the guidelines/evidence. | 4 |
|  |  | Lack of communication/inter-professional collaboration | 2 |
|  |  | Lack of Motivation among provider | 1 |
| 4. | Issues related to Community perception, Knowledge and preference:  ( 8 Studies) | Lack of acceptability and negative attitude due to stigma associated due to its abortion inducing properties | 3 |
|  |  | Hindrance from relatives in taking misoprostol | 1 |
|  |  | Lack of community awareness and knowledge for misoprostol | 2 |
|  |  | Lack of health seeking behavior | 2 |
|  |  | Lack of preference to Medical Abortion using misoprostol | 1 |
|  |  | Lack of access to misoprostol due socio-economic, Ethnic and cultural barriers | 2 |
|  |  | Patients’ lack of trust of lower-cadre health workers | 1 |
|  |  | Disparities in service utilization between rural and urban | 1 |
| 5 | Barriers or gaps related to Health information system (1 Study) | Lack of national reporting on HMIS on use of uterotonics. | 1 |
|  |  | Gaps in inclusion of maternal health indicators in national data | 1 |
| 6 | Barriers or gaps related to cost of medicine  (2 Studies) | Paying for medicine is a bottle neck to improve coverage despite to be inexpensive | 2 |
|  |  | Financial constraints in term of training TBA’s, cost of drug | 1 |

## **Table S5: Quality Assessment of included studies:**

| Study | Explicit Aim | Sample size justification or adequate | Justification sample representative of population | Inclusion and exclusion criteria stated | Response rate and drop out specified | Data adequately described | Statistical significance assessed | Discussion of generalizability | Reliability and validity measures justified | Null findings interpreted | Total |
| --- | --- | --- | --- | --- | --- | --- | --- | --- | --- | --- | --- |
| Osur, J., Baird, T. L., Levandowski, B. A., Jackson, E., & Murokora, D. (2013). Implementation of misoprostol for post abortion care in Kenya and Uganda: a qualitative evaluation. *Global HealthAction*, *6*,10.3402/gha.v6i0.19649.<http://doi.org/10.3402/gha.v6i0.19649> | Y | Y | Y | Y | Y | Y | N | Y | N | N | 7 |
| Ansari, N., Zainullah, P., Kim, Y. M., Tappis, H., Kols, A., Currie, S., Stekelenburg, J. (2015). Assessing post-abortion care in health facilities in Afghanistan: a cross-sectional study. *BMC Pregnancy and Childbirth*, *15*, 6. <http://doi.org/10.1186/s12884-015-0439-x> | Y | Y | Y | Y | Y | Y | Y | Y | Y | Y | 10 |
| Deepak, N. N., Mirzabagi, E., Koski, A., & Tripathi, V. (2013). Knowledge, Attitudes, and Practices Related to Uterotonic Drugs during Childbirth in Karnataka, India: A Qualitative Research Study. *PLoS ONE*, *8*(4), e62801. <http://doi.org/10.1371/journal.pone.0062801> | Y | Y | Y | Y | N | Y | N | Y | Y | N | 7 |
| Mir, A. M., Wajid, A., & Gull, S. (2012). Helping rural women in Pakistan to prevent postpartum hemorrhage: A quasi experimental study.*BMC Pregnancy and Childbirth*,*12*,120.<http://doi.org/10.1186/1471-2393-12-120> | Y | Y | Y | Y | Y | Y | Y | Y | N | Y | 9 |
| Report on Management of Postpartum Hemorrhage - Findings from a survey with 69 FIGO Member Associations, 2016 | Y | N | N | Y | N | Y | Y | Y | N | Y | 6 |
| Prata, N., Bell, S., & Quaiyum, M. A. (2014). Modeling maternal mortality in Bangladesh: the role of misoprostol in postpartum hemorrhage prevention.*BMC Pregnancy and Childbirth*, *14*, 78. <http://doi.org/10.1186/1471-2393-14-78> | Y | N | N | Y | N | Y | Y | Y | Y | Y | 7 |
| Prata, N., Bell, S., Holston, M., & Quaiyum, M. A. (2014). Is attendant at delivery associated with the use of interventions to prevent postpartum hemorrhage at home births? The case of Bangladesh. *BMC Pregnancy and Childbirth*, *14*, 24. <http://doi.org/10.1186/1471-2393-14-24> | Y | Y | Y | Y | Y | Y | Y | Y | N | N | 8 |
| Starrs, A., & Winikoff, B. (2012). Misoprostol for postpartum hemorrhage: Moving from evidence to practice. International Journal of Gynecology & Obstetrics, 116(1), 1-3. | Y | N | N | Y | Y | Y | N | Y | Y | N | 6 |
| Smith, J. M., Currie, S., Cannon, T., Armbruster, D., & Perri, J. (2014). Are national policies and programs for prevention and management of postpartum hemorrhage and preeclampsia adequate? A key informant survey in 37 countries. *Global Health, Science and Practice*, *2*(3), 275–284. <http://doi.org/10.9745/GHSP-D-14-00034> | Y | Y | Y | N | Y | Y | N | Y | Y | N | 7 |
| Aniteye, P., & Mayhew, S. H. (2013). Shaping legal abortion provision in Ghana: using policy theory to understand provider-related obstacles to policy implementation. *Health Research Policy and Systems*, *11*, 23. <http://doi.org/10.1186/1478-4505-11-23> | Y | Y | Y | Y | Y | Y | N | N | Y | N | 7 |
| Haver, J., Ansari, N., Zainullah, P., Kim, Y. M., & Tappis, H. (2016). Misoprostol for Prevention of Postpartum Hemorrhage at Home Birth in Afghanistan: Program Expansion Experience. Journal of Midwifery & Women’s Health. | Y | Y | Y | Y | N | Y | Y | Y | Y | N | 8 |
| Pronyk, P. M., Nemser, B., Maliqi, B., Springstubb, N., Sera, D., Karimov, R., & Leads, U. A. (2016). The UN Ycommission on Life Saving Commodities 3 years on: global progress update and results of a multicountry assessment. *The Lancet Global Health*, *4*(4), e276- | Y | N | Y | Y | N | Y | Y | Y | N | N | 6 |
| Report on Prevention of Postpartum Hemorrhage in Rural Ethiopia, prepared by Technology Transfer and Research Translation Directorate at the Ethiopian Health and Research Institute, March, 2012 | Y | N | N | Y | Y | N | Y | Y | Y | N | 6 |
| Ritchie, L. M. P., Khan, S., Moore, J. E., Timmings, C., van Lettow, M., Vogel, J. P. & Uka, S. (2016). Low-and middle-income countries face many common barriers to implementation of maternal health evidence products. *Journal of clinical epidemiology*. | Y | Y | N | Y | N | Y | N | Y | Y | N | 6 |
| Spangler, S. A., Gobezayehu, A. G., Getachew, T., & Sibley, L. M. (2014). Interpretation of National Policy Regarding Community‐Based Use of Misoprostol for Postpartum Hemorrhage Prevention in Ethiopia: A Tale of Two Regions. Journal of Midwifery & Women’s Health, 59(s1), S83-S90. | Y | N | Y | Y | N | Y | Y | Y | Y | N | 7 |
| Casey, S.E., Chynoweth, S, K., Cornier, N., Gallagher, M.C., & Wheeler, E.E. (2015) Progress and gaps in reproductive health services in three humanitarian settings: mixed-methods case studies, *Conflict & Health*, 9(1), 1. | Y | N | Y | Y | Y | Y | Y | Y | Y | N | 8 |
| Khan, S., Timmings, C., Vogel, J., Islam, S., Puchalski,L., & Straus, S. E. Understanding Barriers and Facilitators to Implementation of Maternal Health Guidelines in Tanzania: A Great Network Research Activity. | Y | Y | Y | Y | N | Y | N | Y | N | N | 6 |
| Ejembi, C., Shittu, O., Moran, M., Adiri, F., Oguntunde, O., Saadatu, B., & Williams, N. (2014). Community-level distribution of misoprostol to prevent postpartum hemorrhage at home births in northern Nigeria. African journal of reproductive health, 18(2), 166-175. | Y | Y | N | Y | Y | Y | N | Y | Y | N | 7 |
| Moore, J. E., Uka, S., Vogel, J. P., Timmings, C., Rashid, S., Gülmezoglu, A. M., & Straus, S. E. (2016). Navigating barriers: two-year follow up on recommendations to improve the use of maternal health guidelines in Kosovo. BMC Public Health, 16(1), 987. | Y | Y | N | Y | Y | Y | N | Y | Y | N | 8 |
| Starrs, A., & Winikoff, B. (2012). Misoprostol for postpartum hemorrhage: Moving from evidence to practice. International Journal of Gynecology & Obstetrics, 116(1), 1-3. | Y | N | N | Y | Y | Y | N | Y | Y | N | 6 |

(Mizra & Jenkins, 2004).

## **Table S6: Barriers or gaps identified from included articles**

| **S.No** | **Health System Building Blocks/Thematic Areas** | **Sub-Themes for identified gaps or barriers** | **Number of studies identified similar gap/barriers** | **Study Codes** |
| --- | --- | --- | --- | --- |
| 1 | Barriers or gaps related to Leadership, governance, Guidelines and Policy related  (12 Studies) | Lack of national policies and guidelines for MPAC or PPH | 2 | SR-001, SR-020 |
|  |  | No Registration of misoprostol Specific for PAC or PPH | 3 | SR-001, SR-005, SR-007 |
|  |  | Fear and apprehensions related to its use | 3 | SR-007, ,SR-016, SR-018 |
|  |  | Misoprostol not included in National list of essential medicines | 1 | SR-005 |
|  |  | legal restrictions that only permits pregnancy termination to save life of mother | 1 | SR-002 |
|  |  | No or Less Preference to misoprostol | 1 | SR-009 |
|  |  | Lack of integration of misoprostol with Basic package of health services/ health care resources | 2 | SR-012, SR-015 |
|  |  | Poorly developed Commodity security strategies. | 1 | SR-013 |
|  |  | Rudimentary or absent Transportation and communication system. | 2 | SR-014, SR-018 |
|  |  | Lack of ability to implement and monitor implementation and current practices. | 1 | SR-015 |
|  |  | Lack of trust between clinicians and policy makers | 1 | SR-015 |
|  |  | Gaps in pre-service medical and midwifery education program curriculum. | 1 | SR-002 |
|  |  | Technical inconsistencies and ambiguity in guidelines and protocols- incomplete and out dated. | 2 | SR-007, SR-009 |
|  |  | Lack of communication or awareness of existing policy | 2 | SR-016, SR-018 |
| 2 | Barriers or gaps related to Health Service Delivery, and availability and access to essential medicine  (10 Studies) | Lack of Access to misoprostol | 2 | SR-009, SR-018 |
|  |  | PAC services not available | 1 | SR-017 |
|  |  | Issues related to inconsistencies in supplies/ fragmented supply chain and distribution | 8 | SR-001, SR-002, SR-009, SR-014 SR-015, SR-016, SR-017, SR-018 |
|  |  | Lack of Supervision/Monitoring capacity | 1 | SR-013 |
|  |  | Reduced institutional delivery | 1 | SR-003 |
|  |  | Lack of provider preference of Medical Abortion using misoprostol | 1 | SR-017 |
| 3 | Barriers or gaps related to Health Workforce  (14 studies) | Lack of Knowledge & Skills (Technical & non –technical) of providers | 3 | SR-002, SR-003, SR-017 |
|  |  | Lack of training and training capacity of providers | 2 | SR-015,SR-018 |
|  |  | scarcity of staff or inadequate staffing (SBA’s, TBA’s, CHW’s) | 6 | SR-001, SR-008 , SR-012, SR-014, SR-017, SR-018 |
|  |  | Fear, apprehensions and doubt related to misoprostol | 5 | SR-007, SR-010, SR-014, SR-16, SR-018 |
|  |  | Negative or Judgmental attitude of providers | 1 | SR-010 |
|  |  | Limited scope of practice of midwifes | 1 | SR-009 |
|  |  | Lack of awareness/Clarity of the guidelines/evidence. | 4 | SR-005, SR-015, SR-018, SR-020 |
|  |  | Lack of communication/inter-professional collaboration | 2 | SR-015, SR-020 |
|  |  | Lack of Motivation among provider | 1 | SR-020 |
| 4. | Issues related to Community perception, Knowledge and preference:  ( 8 Studies) | Lack of acceptability and negative attitude due to stigma associated due to its abortion inducing properties | 3 | SR-002, SR-004, SR-017 |
|  |  | Hindrance from relatives in taking misoprostol | 1 | SR-004 |
|  |  | Lack of community awareness and knowledge for misoprostol | 2 | SR-004, SR-006, |
|  |  | Lack of health seeking behavior | 2 | SR-015, SR-018 |
|  |  | Lack of preference to Medical Abortion using misoprostol | 1 | SR-002 |
|  |  | Lack of access to misoprostol due socio-economic, Ethnic and cultural barriers | 2 | SR-015, SR-019 |
|  |  | Patients’ lack of trust of lower-cadre health workers | 1 | SR-015 |
|  |  | Disparities in service utilization between rural and urban | 1 | SR-012 |
| 5 | Barriers or gaps related to Health information system (1 Study) | Lack of national reporting on HMIS on use of uterotonics. | 1 | SR-009 |
|  |  | Gaps in inclusion of maternal health indicators in national data | 1 | SR-009 |
| 6 | Barriers or gaps related to cost of medicine  (2 Studies) | Paying for medicine is a bottle neck to improve coverage despite to be inexpensive | 2 | SR-009, SR-014 |
|  |  | Financial constraints in term of training TBA’s, cost of drug | 1 | SR-014 |

## **Table S7: PRISMA Checklist**

| Section/topic | # | Checklist item | Reported on page # |
| --- | --- | --- | --- |
| TITLE | | | |
| Title | 1 | Identify the report as a systematic review, meta-analysis, or both | Pg. 01 |
| ABSTRACT | | | |
| Structured summary | 2 | Provide a structured summary including, as applicable: background; objectives; data sources; study eligibility criteria, participants, and interventions; study appraisal and synthesis methods; results; limitations; conclusions and implications of key findings; systematic review registration number | Pg. 07 |
| INTRODUCTION | | | |
| Rationale | 3 | Describe the rationale for the review in the context of what is already known. | Pg. 09 |
| Objective | 4 | Provide an explicit statement of questions being addressed with reference to participants, interventions, comparisons, outcomes, and study design (PICOS) | Pg. 10 |
| METHODS | | | |
| Protocol and Registration | 5 | Indicate if a review protocol exists, if and where it can be accessed (e.g., Web address), and, if available, provide registration information including registration number. | Not applicable |
| Eligibility criteria | 6 | Specify study characteristics (e.g., PICOS, length of follow-up) and report characteristics (e.g., years considered, language, publication status) used as criteria for eligibility, giving rationale | Pg. 17-18 |
| Information sources | 7 | Describe all information sources (e.g., databases with dates of coverage, contact with study authors to identify additional studies) in the search and date last searched. | Pg. 17 |
| Search | 8 | Present full electronic search strategy for at least one database, including any limits used, such that it could be repeated. | Pg. 16 |
| Study selection | 9 | State the process for selecting studies (i.e., screening, eligibility, included in systematic review, and, if applicable, included in the meta-analysis). | Pg.16-17 |
| Data collection process | 10 | Describe method of data extraction from reports (e.g., piloted forms, independently, in duplicate) and any processes for obtaining and confirming data from investigators. | Pg.18 |
| Data Items | 11 | List and define all variables for which data were sought (e.g., PICOS, funding sources) and any assumptions and simplifications made. | Annexure 4, pg. 50 |
| Risk of bias in individual studies | 12 | Describe methods used for assessing risk of bias of individual studies (including specification of whether this was done at the study or outcome level), and how this information is to be used in any data synthesis. | Not applicable |
| Summary measures | 13 | State the principal summary measures (e.g., risk ratio, difference in means). | Not applicable |
| Synthesis of results | 14 | Describe the methods of handling data and combining results of studies, if done, including measures of consistency (e.g., I2) for each meta-analysis. | Pg. 25 |
| Risk of bias across studies | 15 | Specify any assessment of risk of bias that may affect the cumulative evidence (e.g., publication bias, selective reporting within studies). | Pg. 38 |
| Additional analysis | 16 | 16 Describe methods of additional analyses (e.g., sensitivity or subgroup analyses, meta-regression), if done, indicating which were pre-specified. | Not applicable |
| RESULTS | | | |
| Study selection | 17 | Give numbers of studies screened, assessed for eligibility, and included in the review, with reasons for exclusions at each stage, ideally with a flow diagram. | Pg. 22 & 23 |
| Study characteristics | 18 | For each study, present characteristics for which data were extracted (e.g., study size, PICOS, follow-up period) and provide the citations. Risk of bias within studies | Pg. 50 to pg. 90 |
| Risk of Bias with studies | 19 | Present data on risk of bias of each study and, if available, any outcome level assessment (see item 12). | Not applicable |
| Result of Individual studies | 20 | For all outcomes considered (benefits or harms), present, for each study: (a) simple summary data for each intervention group (b) effect estimates and confidence intervals, ideally with a forest plot. | Pg.23-33  a & b not applicable |
| Synthesis of results | 21 | Present results of each meta-analysis done, including confidence intervals and measures of consistency. | Not applicable , however findings are synthesized on pg., 28 |
| Risk of Bias across studies | 22 | Present results of any assessment of risk of bias across studies (see Item 15) | Not done |
| Additional analysis | 23 | Give results of additional analyses, if done (e.g., sensitivity or subgroup analyses, meta-regression [see Item 16]). | Not applicable |
| DISCUSSIONS | | | |
| Summary of evidences | 24 | Summarize the main findings including the strength of evidence for each main outcome; consider their relevance to key groups (e.g., healthcare providers, users, and policy makers). | Pg. 34-37 |
| Limitations | 25 | Discuss limitations at study and outcome level (e.g., risk of bias), and at review-level (e.g., incomplete retrieval of identified research, reporting bias). | Pg.38 |
| Conclusions | 26 | Provide a general interpretation of the results in the context of other evidence, and implications for future research. | Pg.39 |
| FUNDING | | | |
| Funding | 27 | Describe sources of funding for the systematic review and other support (e.g., supply of data); role of funders for the systematic review | Not applicable |

## **Table S8: Filled data Extraction Forms:**

### **Table S8.1**

| **Name of Journal**  Global Health Action | **Date of review**  24^th^ Feb, 2013 | **Year of Publication**  2013 | **Unique form ID:**  SR-001 |
| --- | --- | --- | --- |
| **Title of publication** | Implementation of misoprostol for post abortion care in Kenya and Uganda: a qualitative evaluation. | | |
| **Author(s)** | Joachim Osur, Traci L. Baired, Brooke A. Levandowski, Emily Jackson and Daniel Murokora. | | |
| **Study settings** | Country: Kenya and Uganda  Rural/Urban: Urban  Public/Private facility: both | | |
| **Study Design** | Qualitative study | | |
| **Data collection Methodology and tool** | Qualitative, utilizing In depth interviews | | |
| **Targeted Audience** | Service providers, Health Facility Managers, MoH officials, NGO staff involved in program implementation | | |
| **Variable/ domain of interest** | 1. Identified supportive and inhibitive policies 2. Assessed provider satisfaction 3. Provider impression for client’s satisfaction | | |
| **What implementation gaps or barriers were identified?** | 1. Lack of national policies and guidelines for MPAC. 2. Registration of misoprostol specifically for PAC 3. Procuring misoprostol and maintain adequate supplies. 4. Inadequate staffing of facilities with providers trained in MPAC | | |
| **What are the advantages mentioned of implementing misoprostol for PPH or PAC?** | 1. Acceptability of women to MPAC 2. Task shifting of MPAC provision to midlevel providers in a rational manner 3. Offer women greater privacy, reduced cost, option for non-invasive treatment for incomplete abortion. | | |
| **What is/ are the of limitation/s of this study** | 1. Single interviewer conducting interview country wide 2. Baseline service delivery data prior to implementation and client’s satisfaction data from women receiving MPAC services were not gathered. | | |
| **If this study is being excluded give reason?** | Not excluded | | |
| **Conclusion** | Despite MPAC is cost-effective, simple and scientifically proven intervention services are not available where needed. In light of gaps highlighted this study suggest following Facilitating factors include; Enabling environment, adequate training and on-going support of providers, ease of provision of MPAC, and linking MPAC with maternal mortality reduction goals , for successful and effective implementation of MPAC. | | |
| **Recommendations/ Policy implications** | 1. Registration of misoprostol for treatment of incomplete 2. Accurately estimating demand for misoprostol 3. Creating a formal plan to maintain supplies of misoprostol | | |

### **Table S8.2**

| **Name of Journal**  BMC pregnancy and child birth | **Date of review**  7^th^ July, 2014 | **Year of Publication**  2015 | **Unique form ID:**  SR-002 |
| --- | --- | --- | --- |
| **Title** **of publication** | Assessing post abortion care in health facilities in Afghanistan : a cross sectional study | | |
| **Author(s)** | Nasratullah Ansari, Partamin Zainullah, Young Mi kim, Hannah Tappis, Adrienne Kols, Sheena Currie, Jamie Haver, Jos van Roosmalen, Jacqueline EW Broerse and Jelle Stekelenburg | | |
| **Study setting** | Country: Afghanistan  Rural/Urban: Across Afghanistan  Public/Private facility: Both | | |
| **Study Design** | A cross-sectional study | | |
| **Data collection Methodology and tool** | Quantitative methodology, using Averting Maternal Death and Disability Program Needs Assessment Toolkit and National Midwifery education assessment tools | | |
| **Targeted Audience** | Female doctors and midwives | | |
| **Variable/ domain of interest** | 1. Caseload, supplies, equipment and staffing 2. Provider knowledge and skills 3. Variables associated with PAC knowledge and skills | | |
| **What implementation gaps or barriers were identified?** | 1. Supplies and equipment needed for PAC 2. Insufficient knowledge, capacity and skills of service providers 3. Limited coverage of PAC in pre-service medical and midwifery education program 4. Stigma associated with it due to its abortion inducing properties 5. legal restrictions that only permits pregnancy termination to save life of mother 6. Preferring MVA over Medical Abortion (MA) using misoprostol. 7. Use for paracetamol for post-procedure pain instead of misoprostol | | |
| **What are the advantages mentioned in implementing misoprostol for PPH or PAC?** | Cost-effective, heat stable | | |
| **What is/ are the of limitation/s of this study** | 1. Lack of access to other EmNOC facilities due to security concerns 2. Clinical simulation with anatomical models may not depict actual performance. 3. Sampling strategy underrepresent providers | | |
| **If this study is being excluded give reason?** | N/A | | |
| **Conclusion** | Afghanistan generally have supplies and equipment needed for PAC but the capacity of service providers to deliver PAC is limited | | |
| **Recommendations/policy Implications** | 1. Training of all midwives and doctors in PAC through integration of PAC training package to pre-service education curriculum. 2. Treatment of retained products of conception with misoprostol 3. Improving contraceptive counselling trainings for skilled birth attendants (SBAs) and increasing access to multiple contraceptive methods on sites 4. National policies should be updated in line with international standards and training packages revised accordingly. 5. PAC service information should be integrated into National HMIS and national monitoring checklist. | | |

### **Table S8.3**

| **Name of Journal**  PLOS ONE | **Date of review**  Sept, 24, 2012 | **Year of Publication**  **2013** | **Unique form ID:**  SR-003 |
| --- | --- | --- | --- |
| **Title** **of publication** | Knowledge, attitudes and practices related to uterotonics drugs during childbirth in Karnataka, India: A Qualitative research study | | |
| **Author(s)** | Nitya Nand Deepak, Ellie Mirzabagi, Alissa Koski, Vandana Tripathi | | |
| **Study setting** | Country: India  Rural/Urban: mixed  Public/Private facility: both | | |
| **Study Design** | Exploratory | | |
| **Data collection Methodology and tool** | Qualitative method using semi-structure in-depth interviews | | |
| **Targeted Audience** | Physicians, Nurses, TBAs, Unlicensed village doctors. Pharmacists, women who delivered within six months prior and mothers is law with at least one grand child | | |
| **Variable/ domain of interest** | Knowledge attitude and practices of formal and informal service providers and community members | | |
| **What implementation gaps or barriers were identified?** | 1. Misconception and knowledge gap of informal providers and community members (they belief increase pain is needed to facilitate delivery which is achieved by using oxytocin injection). 2. Inconsistency of provider knowledge related to its dosage, required monitoring, and its administration. 3. Reduced institutional deliveries and skilled attendance at birth | | |
| **Wha89t are the advantages mentioned of implementing misoprostol for PPH or PAC?** | Not mentioned | | |
| **What is/ are the of limitation/s of this study** | 1. Potential recall bias. 2. It is not possible to conduct multiple interviews with subjects, which would enabled probes on specific topics emerged after interview with other cadres | | |
| **If this study is being excluded give reason?** | Not Excluded | | |
| **Conclusion** | This study documented common use of uterotonics, particularly oxytocin. Much described use is inappropriate in the context of global and national guidance for administration of uterotonics. | | |
| **Recommendations/ Policy Implications** | 1. Applying approaches [client oriented, provider- efficient services (cope) , partnership defined quality (PDQ) that bring community and provider together to share their perception of highest quality of care. 2. Incorporating pre-service training using updated curriculum in light of current guidance. 3. Providing in-service/refresher training to align knowledge with current SBA guidelines. 4. Developing education and behavior change communication (BCC) materials for all relevant cadres and community members to ensure its appropriate use, dosage, adverse effects and reactions. | | |

### **Table S8.4**

| **Name of Journal**  BMC Pregnancy & Child birth | **Date of review**  27/10/2012 | **Year of Publication**  **2012** | **Unique form ID:**  SR-004 |
| --- | --- | --- | --- |
| **Titleof publication** | Helping rural women in Pakistan to prevent postpartum hemorrhage: A quasi experimental study | | |
| **Author(s)** | Ali Muhammad Mir, Abdul Wajid, Sadaf Gull | | |
| **Study setting** | Country: Pakistan  Rural/Urban: Rural  Public/Private facility: n/a | | |
| **Study Design** | A quasi experimental | | |
| **Data collection Methodology and tool** | Quantitative method using structured questionnaire | | |
| **Targeted Audience** | Pregnant women , intended to deliver at home and would be delivering during study period | | |
| **Variable/ domain of interest** | 1. Feasibility of distribution and administration of misoprostol in home based settings. 2. Assess acceptability and use of misoprostol | | |
| **What implementation gaps or barriers were identified?** | 1. Hindrance from relatives in taking misoprostol (opposing view). 2. Lack of knowledge of study participants regarding timings of taking misoprostol and appropriate timings for referrals | | |
| **What are the advantages mentioned of implementing misoprostol for PPH or PAC?** | 1. One-drug regimen, easy administration and stability as compared to injectable oxytocin. 2. Reduction in demand of referral due to misoprostol ingestion. | | |
| **What is/ are the of limitation/s of this study** | PPH rate could be underestimated as study didn’t measure actual blood loss. | | |
| **If this study is being excluded give reason?** | Not excluded | | |
| **Conclusion** | Self-administration of misoprostol in home based setting is feasible provided proper training of providers and information exchange among community members. | | |
| **Recommendations** | Proper counseling and information exchange are recommended for bringing new practices in low –resource settings. | | |

### **Table S8.5**

| **Name of Journal**  N/A | **Date of review**  ----- | **Year of Publication**  **2016** | **Unique form ID:**  SR-005 |
| --- | --- | --- | --- |
| **Title of publication** | Management of Postpartum Hemorrhage - Findings from a survey with 69 FIGO Member Associations | | |
| **Author(s)** | International Federation of Gynecology and Obstetrics | | |
| **Study setting** | Country: developing countries  Rural/Urban: both  Public/Private facility: n/a | | |
| **Study Design** | A web-based survey | | |
| **Data collection Methodology and tool** | survey was developed and sent out by email to 130 FIGO Member Associations | | |
| **Targeted Audience** | Members of FIGO association | | |
| **Variable/ domain of interest** | National guidelines for PPH, and inclusion of key PPH medicines on national EMLs as well as any challenges to implementing evidence-based practice. | | |
| **What implementation gaps or barriers were identified?** | 1. Healthcare providers don’t know about guidelines. 2. Misoprostol not included in National EML. 3. No guideline for misoprostol specific for PPH. 4. No registration of misoprostol specific for PPH | | |
| **What are the advantages mentioned of implementing misoprostol for PPH or PAC?** | 1. Safe and clinically effective if administered appropriately. 2. Cost-effective intervention | | |
| **What is/ are the of limitation/s of this study** | This survey was limited in that it did not ask about which healthcare providers were able to give key medicines, and was directed at the guidelines for obstetricians and gynecologists rather than other healthcare providers | | |
| **If this study is being excluded give reason?** | Not excluded | | |
| **Conclusion** | Having comprehensive, evidence-based guidelines on PPH and having misoprostol listed in national EMLs are key interventions which must be in place for a country to address the major cause of maternal mortality and morbidity. However, these alone will be ineffective unless there are also supportive policy and programs in place, broad dissemination and training of the guidelines, and full drug availability throughout all health facilities | | |
| **Recommendations / Policy Implications** | 1. Need for supportive policy and program in place for its complete implementation. 2. Broad dissemination and training of the guidelines. 3. Need to include misoprostol in National EML where it won’t yet include. | | |

### **Table S8.6**

| **Name of Journal**  BMC pregnancy and childbirth | **Date of review**  2-Aug-2013 | **Year of Publication**  2014 | **Unique form ID:**  SR-006 |
| --- | --- | --- | --- |
| **Title** **of publication** | Modeling maternal mortality in Bangladesh: the role of misoprostol in postpartum hemorrhage prevention | | |
| **Author(s)** | Ndola Prata, Suzanne Bell, Md Abdul Quaiyum | | |
| **Study setting** | Country: Bangladesh  Rural/Urban: Rural  Public/Private facility: n/a | | |
| **Study Design** | Operational research | | |
| **Data collection Methodology and tool** | Qualitative, verbal autopsy questionnaire | | |
| **Targeted Audience** | Pregnant Women | | |
| **Variable/ domain of interest** | Safety, feasibility and acceptability of low-level providers in administering misoprostol for home based delivery. | | |
| **What implementation gaps or barriers were identified?** | 1. Little knowledge of community regarding causes of PPH and using misoprostol in such conditions. | | |
| **What are the advantages mentioned of implementing misoprostol for PPH or PAC?** | 1. Has potential to prevent PPH attributable maternal mortality at community level in low resource settings. 2. Cost effective intervention | | |
| **What is/ are the of limitation/s of this study** | 1. Alternative explanations could also be used to explain the difference in mortality among those who did and did not take misoprostol possibly due to ecological association. 2. Another limitation was the use of verbal autopsy to determine cause of death which perhaps result in differential misclassification of cause of death. 3. Assuming the constant rate of PPH associated mortality across population | | |
| **If this study is being excluded give reason?** | Not excluded | | |
| **Conclusion** | Prophylactic use of misoprostol at home birth may contribute in averting maternal deaths due to PPH. | | |
| **Recommendations / Policy Implications** | 1. Getting more women to deliver at facility by encouraging early transfer among home births. 2. Increase coverage of misoprostol among women delivering at home. | | |

### **Table S8.7**

| **Name of Journal**  International journal of Gynecology and obstetrics | **Date of review**  ----- | **Year of Publication**  **2012** | **Unique form ID:**  SR-007 |
| --- | --- | --- | --- |
| **Title of publication** | Misoprostol for postpartum hemorrhage: Moving from evidence to practice | | |
| **Author(s)** | Ann Starss, Beverly Winikoff | | |
| **Study setting** | Country: developing countries  Rural/Urban: both  Facility/community based: community based  Public/Private facility: n/a | | |
| **Study Design** | Special communication | | |
| **Data collection Methodology and tool** | Not mentioned | | |
| **Targeted Audience** | Nil | | |
| **Variable/ domain of interest** | 1. Safety, feasibility and effectiveness of misoprostol for community or home based settings. | | |
| **What implementation gaps or barriers were identified?** | 1. Association with abortion endanger discomfort and resistance among policy makers and providers. 2. No registration of misoprostol for other than management of gastric ulcer. 3. Lack of clear and consistent usage guidance often outdated, non-evidence based protocol in practice. 4. Fear of its promotion, it may deter women from seeking care at facilities with skilled providers. 5. It is perceived that if misoprostol is widely available, it will be repurposed for used in medical abortion, thus increasing number of abortions. | | |
| **What are the advantages mentioned of implementing misoprostol for PPH or PAC?** | 1. Safe and clinically effective if administered appropriately. 2. Cost-effective intervention | | |
| **What is/ are the of limitation/s of this study** | Not given. | | |
| **If this study is being excluded give reason?** | Not excluded | | |
| **Conclusion** | Current evidence support misoprostol use for community and home based setting in absence of parenteral uterotonics for PPH prevention in low and middle income countries.  Policy makers and decision makers are required to have farsighted view by weighing up potential health benefits , cost-effectiveness and feasibility between implementing misoprostol for PPH and other interventions like prevention of anemia, horizontal health system strengthening etc. to improve poor maternal health outcomes | | |
| **Recommendations / Policy Implications** | 1. Practitioner, policy makers and advocators work together to ensure that concerns about misoprostol use for other indications don’t lead to limitations on its availability. 2. Evidence based guidelines for the use of misoprostol for PPH need to be put in place at the national level and this must be supplemented by training and ongoing education of providers. Since this drug can be used for multiple indications, training must include clear dosing information, including the routes and precautions associated. | | |

### **Table: S8.8**

| **Name of Journal**  BMC Pregnancy and childbirth | **Date of review**  6 Jan 2014 | **Year of Publication**  **2014** | **Unique form ID:**  SR-008 |
| --- | --- | --- | --- |
| **Title** **of publication** | Is attendant at delivery associated with the use of interventions to prevent postpartum hemorrhage at home births? The case of Bangladesh | | |
| **Author(s)** | Ndola Prata, Suzanne Bell, Md Abdul Quaiyum, Martine Holston | | |
| **Study setting** | Country: Bangladesh  Rural/Urban: Rural  Facility/community based: community based.  Public/Private facility: n/a | | |
| **Study Design** | Cross-sectional | | |
| **Data collection Methodology and tool** | Quantitatively, using Ante-natal care (ANC) cards | | |
| **Targeted Audience** | Women who deliver at home without a skilled providers and use misoprostol and delivery mat. | | |
| **Variable/ domain of interest** | Safety, feasibility and acceptability of scaling up community based provision of misoprostol. | | |
| **What implementation gaps or barriers were identified?** | Inadequate Coverage of SBAs and TBA’s | | |
| **What are the advantages mentioned of implementing misoprostol for PPH or PAC?** | Use of misoprostol is safe, feasible for home based used and effective intervention | | |
| **What is/ are the of limitation/s of this study** | 1. All data were self-reported by women may lead to social desirability bias. 2. Direct observation was not possible. 3. Cross-sectional data, thus can’t claim causality and alternative explanations of finding | | |
| **If this study is being excluded give reason?** | Not excluded | | |
| **Conclusion** | TBAs can have significant impact on effective utilization of interventions. ANC visit could be the crucial point of contact for information transfer and message reinforcement regarding prevention of PPH | | |
| **Recommendations/ Policy Implications** | Strengthening recruitment of women for ANC by encourage attendance at multiple visits and training of existing TBAs will increase utilization among women | | |

### **Table: S8.9**

| **Name of Journal**  Global health: Science and practice | **Date of review**  27 May, 2014 | **Year of Publication**  **2014** | **Unique form ID:**  SR-009 |
| --- | --- | --- | --- |
| **Titleof publication** | Are national policies and programs for prevention and management of postpartum hemorrhage and preeclampsia adequate? A key informant survey in 37 countries | | |
| **Author(s)** | Jeffery Micheal Smith, Sheena Currie, Tirza Cannon, Deborah Armnruster, Julia Perri | | |
| **Study setting** | Country: Lower and middle income countries  Rural/Urban: -----  Facility/community/home based: In general  Public/Private facility: ----- | | |
| **Study Design** | Cross-sectional | | |
| **Data collection Methodology and tool** | mixed method using Key informants survey and questionnaire | | |
| **Targeted Audience** | No target specific audience since survey reported on publically available information | | |
| **Variable/ domain of interest** | Policy, training, medication distribution and logistics, National reporting of key maternal indicators and challenges to and opportunities for scale up | | |
| **What implementation gaps or barriers were identified?** | 1. More countries included oxytocin essential medicine list than misoprostol. 2. Inconsistent and limited availability of misoprostol limits implementation of national priorities. 3. Misoprostol use to prevent PPH at home birth have piloted in some countries but far fewer have taken the strategy to scale up. 4. Barriers to access and availability. 5. Paying for medicine is a bottle neck to improve coverage despite to be inexpensive. 6. Technical inconsistencies in national service deliver guidelines (SDG) – incomplete and out dated. 7. Lack of national reporting on HMIS on use of uterotonics. 8. Limited scope of practice of midwifes 9. Gaps in inclusion of maternal health indicators in national data. | | |
| **What are the advantages mentioned of implementing misoprostol for PPH or PAC?** | Misoprostol is lifesaving intervention in low resource setting in absence of skilled birth attendant and in case of non-availability of oxytocin | | |
| **What is/ are the of limitation/s of this study** | 1. Limited access to such information or data. 2. Little information of representatives in stake holder 3. group particularly MOH involvement. 4. Many countries use different terms for same process or activity, certain nuances have been lost. | | |
| **If this study is being excluded give reason?** | Not excluded. | | |
| **Conclusion** | Most countries have essential policies and program elements to manage/prevent PPH and Pre-eclampsia but several gaps including absence of commodities (especially misoprostol), limitation in scope of practice of midwives, gaps in national data systems have hindered efforts to scale-up nationally. National programs need to ensure both adequate coverage and sustainability along with aggressive tracking of progress of national programs. | | |
| **Recommendations and Policy Implications** | 1. Increase support for using misoprostol to prevent PPH at home births to allow greater coverage. 2. Updating national clinical guidelines and EML to be consistent with WHO recommendations for PPH. 3. Revision for health monitoring and reporting system to improve and tract indicators related to maternal health. 4. Clear definition and uniform application of midwifery practice scope. | | |

### **Table: S8.10**

| **Name of Journal**  Health research Policy and systems | **Date of review**  2^nd^ June, 2013 | **Year of Publication**  **2013** | **Unique form ID:**  SR-010 |
| --- | --- | --- | --- |
| **Titleof publication** | Shaping legal abortion provision in Ghana: Using policy theory to understand provider-related obstacles to policy implementation | | |
| **Author(s)** | Patience Aniteye and Susannah H Mayhew | | |
| **Study setting** | Country: Ghana  Rural/Urban: Urban  Facility/community based: In general  Public/Private facility: n/a | | |
| **Study Design** | Qualitative Study | | |
| **Data collection Methodology and tool** | Qualitative , using In depth interview and document analysis | | |
| **Targeted Audience** | Managers, Obstetrician, Gynecologist, pharmacists and midwives | | |
| **Variable/ domain of interest** | Providers attitudes and values in shaping safe abortion service delivery | | |
| **What implementation gaps or barriers were identified?** | 1. Doubt about standard and protocols for abortion care. 2. Perceived lack of administrative support. 3. Judgmental attitudes because of personal, social and structural reasons | | |
| **What are the advantages mentioned of implementing misoprostol for PPH or PAC?** | Misoprostol is safer alternative to clinical abortion could spread favorable attitudes. | | |
| **What is/ are the of limitation/s of this study** | 1. Limited access to such information or data. 2. Little information of representatives in stake holder group particularly MOH involvement. 3. Many countries use different terms for same process or activity, certain nuances have been lost. | | |
| **If this study is being excluded give reason?** | Not excluded. | | |
| **Conclusion** | There is need to ensure proper implementation of policies and publically framing abortion as health issue rather than moral one This study has established that theory –Lipsky’s street level bureaucrats- works well in lower and middle income countries settings to recognize a complex range of influences that providers faced and explain their use of preference and development of coping mechanism that shape their practice of safe abortion service provision. | | |
| **Recommendations/ Policy Implications** | 1. Utility of value clarification workshops had potential to transform the attitudes of service providers related to abortion. 2. Value-clarification exercise needs to be incorporated into service education program. 3. Require strong advocacy efforts towards safe abortion. 4. Proper implementation of Ministry of health policies on safe abortion services. 5. Regular supervision, mentoring and support of practicing and student midwives and other providers is essential along with performance appraisal. | | |

### **Table S8:11**

| **Name of Journal**  Lancet Global health | **Date of review**  - | **Year of Publication**  May, 06, 2014 | **Unique form ID:**  SR-011 |
| --- | --- | --- | --- |
| **Title of publication** | Global causes of maternal death: a WHO analysis | | |
| **Author(s)** | Lale Say, Doris chou, Alison Germmill, Oge Tuncalp, Ann-beth Moller, Jane Daniels, A Metin Gulmezoglu, Marleen Tennerman, Leotine Alkema. | | |
| **Study settings** | Country: Globally  Rural/Urban: both  Health facility/ community based: In general  Public/Private facility: both | | |
| **Study Design** | Situational analysis | | |
| **Data collection Methodology and tool** | government reports and vital registration data | | |
| **Targeted Audience** | Mothers with pregnancy or termination of pregnancy within 42 days of delivery. | | |
| **Variable/ domain of interest** | To identify global causes of maternal deaths | | |
| **What are the advantages mentioned of implementing misoprostol for PPH or PAC?** | Not mentioned | | |
| **What implementation gaps or barriers were identified?** | Barriers or gaps specific to misoprostol implementation were not identified | | |
| **What is/ are the of limitation/s of this study** | 1. Limitation of data set and the method used in the study not allowing for generation of worldwide causes of maternal mortality. 2. Results of the analysis were constrained by the accuracy of data included. 3. Misclassification within maternal death certification leads to misinterpretation of cause of death coding rules. | | |
| **If this study is being excluded give reason?** | Excluded; because no implementation gaps particular to misoprostol or uterotonics were identified in this study. | | |
| **Conclusion** | Accurate and routine information regarding causes of maternal deaths is crucial in implementing interventions and tracking and interpretations of gaps in coverage | | |
| **Recommendations /policy implications** | Accelerated action is needed to improve data acquisition and quality, especially relating to correct attribution of cause of death information | | |

### **Table: S8.12**

| **Name of Journal**  Journal of Midwifery & Women’s Health | **Date of review**  - | **Year of Publication**  April, 2016 | **Unique form ID:**  SR-012 |
| --- | --- | --- | --- |
| **Title of publication** | Misoprostol for Prevention of Postpartum Hemorrhage at Home Birth in Afghanistan: Program Expansion Experience | | |
| **Author(s)** | Jaime Haver, Nasruthullah Ansari, Partamin Zainullah, Young-Mi Kim,  Hannah Tappies | | |
| **Study settings** | Country: Afghanistan  Rural/Urban: both  Health facility/ community based: community based  Public/Private facility: - | | |
| **Study Design** | Before and after design without control group | | |
| **Data collection Methodology and tool** | Baseline and End-line surveys, verbal autopsy questionnaire | | |
| **Targeted Audience** | Pregnant women or post-partum women | | |
| **Variable/ domain of interest** | Misoprostol implementation program expansion experience in term of household services, Community sensitization, health facility intra-partum services | | |
| **What implementation gaps or barriers were identified?** | 1. Lack of integration of advance distribution of misoprostol with Basic package of health services. 2. Scarcity of CHW’s or alternative cadre to reach women without access to existing health services. 3. Disparities in service utilization between rural and urban | | |
| **What are the advantages mentioned of implementing misoprostol for PPH or PAC?** | 1. It helps to address coverage gaps for non-institutional births. 2. Can be administered by less skilled health personnel 3. High efficacy for PPH prevention | | |
| **What is/ are the of limitation/s of this study** | 1. Lack of generalizability, since the implementation districts were selected purposively. 2. Study findings are based on women’s recollection of their births, which may be subject to recall bias. | | |
| **If this study is being excluded give reason?** | Not Excluded | | |
| **Conclusion** | Development and implementation of National strategy for counselling on PPH prevention and expansion of advance distribution of misoprostol for self-administration in home births will lead to increased used of utertonics both in facilities and home-based and decrease in maternal mortality ratio in Afghanistan | | |
| **Recommendations/ policy implications** | 1. Provide enabling environment for further expansion. 2. Advance distribution of misoprostol be integrated with basic Package of health services, with close monitoring to identify any concerns with CHW workload. 3. Train additional number of CHWs as an alternative strategy to reach women without access to existing health services | | |

### **Table S8.13**

| **Name of Journal**  The Lancet Global Health | **Date of review**  - | **Year of Publication**  April, 2016 | **Unique form ID:**  SR-013 |
| --- | --- | --- | --- |
| **Title** **of publication** | The UN Commission on Life Saving Commodities 3 years on: global progress update and results of a multi-country assessment. | | |
| **Author(s)** | Paul M Pronyk, Bennett Nemser, Blerta Maliqi, Nara Springstubb, Diana Sera, Rouslan Karimov. | | |
| **Study settings** | Country: 12 sub- Saharan African countries (that were off-tract to achieve MDG 4& 5) it includes Republic of Congo, Ethopia, Malawi, Nigeria, Senegal, Sierra Leone, Uganda, Tanzania, Cameroon, Mali, Zambia and Kenya.  Rural/Urban: Both  Health facility/ community based: National/Policy level  Public/Private facility: both | | |
| **Study Design** | Situational Analysis | | |
| **Data collection Methodology and tool** | Review of key documents and reference data, interview with key stakeholders | | |
| **Targeted Audience** | Nil | | |
| **Variable/ domain of interest** | 1. Reproductive, neonatal, maternal and Child (RMNCH) Situation analysis. 2. Progress against global milestone. 3. RMNCH engagement process | | |
| **What implementation gaps or barriers were identified?** | 1. Commodity security strategies were often poorly developed. 2. Capacity to monitor the quality and safety of medicines is scarce. 3. Gap in monitoring the distribution of commodities from national warehouse to service delivery site remain a barrier. 4. Fragmented supply chains. | | |
| **What are the advantages mentioned of implementing misoprostol for PPH or PAC?** | Not mentioned | | |
| **What is/ are the of limitation/s of this study** | Not mentioned | | |
| **If this study is being excluded give reason?** | Although this analysis has a focused on 13 lifesaving commodities; misoprostol is one of them, this situation analysis has also identified gaps and barriers, due to these reason this study is not excluded. | | |
| **Conclusion** | Robust and sustained technical support should be made available to strengthen RMNCH investment cases, ease country access to the latest evidence and best practice materials, enhance regional learning to support implementation of nationally defined health priorities. | | |
| **Recommendations /policy implications** | 1. Establishing fast tract system to accelerate product registration. 2. Generating means and materials to support advocacy, demand generation and health worker training. 3. Use of standard metrics to track commodity flow 4. Establishment of financing mechanisms to underwrite timely domestic commodity procurement. 5. Efforts to improve regulatory efficiency through establishing product standards, harmonization of guidelines, and support for joint inspection. 6. Enhance post market surveillance and strengthen pharmaco-vigilance. | | |

### **Table S8.14**

| **Name of Journal:**  **Nil** | **Date of review**  - | **Year of Publication**  Report drafted on March, 2012 | **Unique form ID:**  SR-014 |
| --- | --- | --- | --- |
| **Title** **of publication** | Prevention of Postpartum Hemorrhage in rural Ethiopia | | |
| **Author(s)** | Ethiopian Health and Research Institute (EHNRI) | | |
| **Study settings** | Country: Ethiopia  Rural/Urban: Rural  Health facility/ community based: both  Public/Private facility: both | | |
| **Study Design** | Situational Analysis | | |
| **Data collection Methodology and tool** | Document review, expert interviews | | |
| **Targeted Audience** | n/a | | |
| **Variable/ domain of interest** | Challenges due to lack of access to uterotonics to prevent PPH in rural Ethiopia. | | |
| **What implementation gaps or barriers were identified?** | 1. Fear of misuse of misoprostol outside the intended purpose for labor induction. 2. Inadequate logistics due to poor road infrastructure, and poor setting for procurement in remote areas. 3. Financial constraints in term of training TBA’s, cost of drug. 4. Shortage of TBA’s | | |
| **What are the advantages mentioned of implementing misoprostol for PPH or PAC?** | 1. Cost effective 2. Thermo stable, safe and high efficacy 3. Low-tech intervention | | |
| **What is/ are the of limitation/s of this study** | - Religious, cultural and gender based disparities were not explored in depth. - Progression and expansion of analysis expanded far beyond the original scope of this investment. | | |
| **If this study is being excluded give reason?** | Not Excluded | | |
| **Conclusion** | Misoprostol must be viewed as one strategy among many that can help to address some of the stubborn challenges faced by health system and facilities that still lack consistent shortage of trained providers and financial constraints. Preventing PPH in rural Ethiopia through distribution of misoprostol is the potential solution that may be more feasible to scale up rapidly. In contrast to improving access to skilled birth attendant at well-established facilities. | | |
| **Recommendations /policy implications** | 1. Strong political commitment from the government for MCH care 2. Requires major funding opportunities and public-private sector collaboration globally. 3. Develop clinical protocols for misoprostol use for PPH prevention and treatment. 4. Resource mobilization through coordination of governmental and non-governmental organizations. 5. Establishing rural community based health insurance system. 6. Trained and deploy more TBA;s based on local need | | |

### **Table S8.15**

| **Name of Journal**  Journal of Clinical Epidemiology | **Date of review**  - | **Year of Publication**  02-02-2016 | **Unique form ID:**  SR-015 |
| --- | --- | --- | --- |
| **Title of publication** | Low-and middle-income countries face many common barriers to implementation of maternal health evidence products. | | |
| **Author(s)** | Lisa M. Puchalski Ritchie, Sobia Khan, Julia E. Moore, Caitlyn Timmings, Monique van Lettow, Joshua P. Vogel, Dina N. Khan, Godfrey Mbaruku, Mwiadhi Mrisho, Kidza Mugerwa, Sami Uka, A. Metin Gulmezolgu, Sharon E. Stratus | | |
| **Study settings** | Country: Low middle income countries (LMIC’s)  Rural/Urban: both  Health facility/ community based: both  Public/Private facility: both | | |
| **Study Design** | Situational analysis | | |
| **Data collection Methodology and tool** | Meeting reports and articles describing projects undertaken in 5 LMIC’s from GREAT Network projects in Kosovo, Myanmar, Malawi Tanzania and Uganda | | |
| **Targeted Audience** | n/a | | |
| **Variable/ domain of interest** | Perceived barriers and facilitators to maternal health evidence implementations related to PPH (in Kosovo), Task shifting (mayanmar), multiple guidelines (Uganda and Tanzania), TB Adherence (Malawi) Multiple guidelines at health system, health facility and community level | | |
| **What implementation gaps or barriers were identified?** | Barriers at Health System level (related to PPH):   1. Lack of supplies especially in small/ rural centres. 2. Lack of ability to smoothly transfer patients/or coordinate care across health system levels. 3. Lack of ability to implement and monitor implementation and current practices. 4. Lack of mechanism to collect high quality data for monitoring and evaluation. 5. Lack of integration/collaboration of health care resources. 6. Lack of trust between clinicians and policy makers   Barriers at Provider Level:   1. Lack of training capacity and time to attend training. 2. Lack of awareness of the guidelines/evidence. 3. Lack of communication/inter-professional collaboration   Barriers at Community level:   1. Cultural practices and lack of health seeking behavior. 2. Ethnic and cultural differences. 3. Patients’ lack of trust of lower-cadre health workers | | |
| **What are the advantages mentioned of implementing misoprostol for PPH or PAC?** | Not mentioned | | |
| **What is/ are the of limitation/s of this study** | 1. Use of project meeting reports and articles as the unit of analysis may have failed to capture some barriers. 2. Assessment were conducted at pre-implementation planning stage for all of the projects many of the barriers represent perceived rather than demonstrated challenges. | | |
| **If this study is being excluded give reason?** | Included | | |
| **Conclusion** | These findings present a start point for developing a framework to guide assessment of barriers to and facilitators of evidence implementation in LMIC health systems. | | |
| **Recommendations /policy implications** | 1. Attention should be given towards policy related barriers through direct engagement of policy makers and other stakeholders groups, is warranted in implementation planning. 2. Understanding the context of barriers as well as prioritization of barriers and implement context specific strategies. | | |

### **Table S8.16**

| **Name of Journal**  Journal of Midwifery & Women’s Health | **Date of review**  Jan 2014 | **Year of Publication**  Feb, 2014 | **Unique form ID:**  SR-016 |
| --- | --- | --- | --- |
| **Title of publication** | Interpretation of National Policy Regarding Community‐Based Use of Misoprostol for Postpartum Hemorrhage Prevention in Ethiopia: A Tale of Two Regions. | | |
| **Author(s)** | Sydney A. Spangler, Abebe Gebramariam Gabezayehu, Tewodros Getachew and Lynn M. Sibley | | |
| **Study settings** | Country: Ethopia  Rural/Urban: Rural  Health facility/ community based: Community based  Public/Private facility: Nil | | |
| **Study Design** | Qualitative study | | |
| **Data collection Methodology and tool** | In-depth interview and survey | | |
| **Targeted Audience** | Government officials | | |
| **Variable/ domain of interest** | Policy context of community based use of misoprostol for prevention of PPH. | | |
| **What implementation gaps or barriers were identified?** | 1. No policy specifies whether community health development agents, TBA’s, or laypersons can handle and administer misoprostol. 2. Confusion regarding the existence of official policy for community based use of misoprostol to prevent PPH. 3. Systematic inconsistencies in drug supply, training and professional (or personal) commitment among lower-level officials and health workers. 4. Confusion about the existence of specific policy for PPH prevention was also apparent. 5. Fear or safety concerns includes administration in the event of an undiagnosed twin (leading to fatal outcome), administration during labour (cause uterine rupture0, and use of unsafe abortion. 6. In response to advance distribution; fears concerning intra-partum administration, undiagnosed multiple gestation, unsafe abortion, medication sharing and possibility of its use after expiry. 7. It is believed that misoprostol will increase the home based delivery and limit the facility based delivery. 8. Lack of communication of policy that exist. | | |
| **What are the advantages mentioned of implementing misoprostol for PPH or PAC?** | 1. Heat stable 2. Doesn’t require injection. 3. Low administration and storage cost. 4. Can be self-administered | | |
| **What is/ are the of limitation/s of this study** | 1. Potential response bias in in-depth interview. 2. Review of transcript by the interviewee to review for overly sensitive response | | |
| **If this study is being excluded give reason?** | Not excluded | | |
| **Conclusion** | Strategy that permits multiple channels of misoprostol distributions (ie, trained community health workers or advance distribution with trained child bearing women and family caregivers) has great potential to increase coverage | | |
| **Recommendations /policy implications** | 1. Assist policy makers in context driven decision making for community based PPH prevention by providing a standardized means of employing existing evidence to evaluate how, where and under what circumstances it make sense to implement this intervention. | | |

### **Table S8.17**

| **Name of Journal**  Conflict and Health | **Date of review** | **Year of Publication**  2 February 2015 | **Unique form ID:**  SR-017 |
| --- | --- | --- | --- |
| **Title of publication** | | Progress and gaps in reproductive health services in three humanitarian settings: mixed-methods case studies | |
| **Author(s)** | | Sara E Casey, Sarah K Chynoweth, Nadine Cornier, Meghan C Gallagher, Erin E Wheeler | |
| **Study settings** | | Country:Sub Saharan African countryinclude Burkina Faso, Democratic republic of Congo (DRC) and Maban country of South Sudan  Rural/Urban: Humanitarian settings  Health facility/ community based: facility based  Public/Private facility: both | |
| **Study Design** | | cross-sectional, mixed methods case study | |
| **Data collection Methodology and tool** | | Quantitatively: Facility assessment checklist, clinical register review, room by room inventory check  Qualitatively: Focus group discussions (FGD’s) with community members | |
| **Targeted Audience** | | Married and un-married women internally displaced person (IDP’s) / refuges and members from host communities | |
| **Variable/ domain of interest** | | To document the availability, quality, and utilization of RH service provision in the three settings and to explore access barriers. | |
| **What implementation gaps or barriers were identified?** | | 1. Dearth of trained staff in providing PAC service 2. Manual vacuum aspiration is the most commonest mean for uterine evacuation in Burkina Faso and DRC 3. FGDs from community revealed negative attitudes toward abortion, which they said conflicted with religious beliefs. 4. Availability of PAC and a full package of FP services were limited in health centers. 5. paucity of drugs as the primary barrier to providing adequate RH care 6. lack of non-technical skills such as poor situation awareness, decision-making, and inter-personal skills including communication and teamwork | |
| **What are the advantages mentioned of implementing misoprostol for PPH or PAC?** | | Not mentioned | |
| **What is/ are the of limitation/s of this study** | | 1. Due to missing and poor quality service statistics, utilization could not be assessed 2. Insecurity and physical obstacles, such as poor roads and rain, were significant barriers across settings and prevented visits to some health facilities 3. Time pressures, high workloads, and coordination challenges among assessment team members resulted in missing data. 4. Possibility of translation error | |
| **If this study is being excluded give reason?** | | Not Excluded | |
| **Conclusion** | | Many health facilities are providing some RH services, there remains an urgent need to address gaps in implementation—in particular safe abortion services—as well as the quality of care, utilization of RH services, and monitoring and evaluation. Minimum quality standards must be met to meet the health needs of affected populations. Yet, only expanding RH service availability is not sufficient. Gaps in management and knowledge, as well as the biases of some providers continue to impede the provision of RH services in humanitarian settings. | |
| **Recommendations /Policy implications** | | 1. Behavior change to increase use of RH services 2. Clinical training should integrate social and cognitive skills that can increase patient safety and streamline service delivery. 3. Supportive supervision should be practiced to help providers improve and maintain these skills and address gaps in service provision 4. community participation through awareness raising of services availability in primary health care is associated with increased utilization as well as improved health outcomes | |

### **Table S8.18**

| **Name of Journal**  Project Report | **Date of review** | **Year of Publication**  Nov, 2014 | **Unique form ID:**  SR-018 |
| --- | --- | --- | --- |
| **Titleof publication** | Understanding barriers and facilitators to implementation of Maternal health guidelines in Tanzania: A great network Research activity | | |
| **Author(s)** | Sobia Khan, Caitlyn Timmings, Dr. Joshua Vogel, Shusmita Islam1, Dr. Lisa Puchalski Ritchie, Dr. Dina Khan , Dr. Julia E. Moore, Dr. A. Metin Gülmezoglu, Dr. Ahmad Makuwani , Dr. Ama Kasalanga , Dr. Godfrey Mbaruku, Dr. Mwifadhi Mrisho and Dr.Sharon E. Straus | | |
| **Study settings** | Country: Tanzania  Rural/Urban: both  Health facility/ community based: Both  Public/Private facility: both | | |
| **Study Design** | Mixed Method study design | | |
| **Data collection Methodology and tool** | Primary data collection occurred during the in-country two-day workshop, this involved focus group discussions, ranking exercise and small and large group discussions | | |
| **Targeted Audience** | Health care administrators, policymakers, non-governmental organization staff, representatives from professional associations, frontline health care providers (e.g., physicians, nurses, and midwives), and researchers/academics. | | |
| **Variable/ domain of interest** | identifying barriers and facilitators to the implementation of the four priority guidelines in Tanzania | | |
| **What implementation gaps or barriers were identified?** | *Barriers at Health system level*   1. Limited access to drugs and challenges in the drug distribution system were identified as barriers to the successful implementation. 2. A shortage of health care providers was described as a barrier experienced throughout the health care system in Tanzania 3. Lack of ability to smoothly transfer patients/or coordinate care across health system levels.   *Barriers at Provider Level:*   1. fear that misoprostol can be misused for abortion: 2. inadequacies in the training curriculum may contribute to lack of compliance with guideline 3. Lack of awareness of the existence of guidelines by health care workers   Barriers at Community level:   1. Limited health-seeking behavior and patient preference for care | | |
| **What are the advantages mentioned of implementing misoprostol for PPH or PAC?** | Heat stable, easy to administered | | |
| **What is/ are the of limitation/s of this study** | 1. Data were collected from a small sample of participants that may not be representative of the entire population working in the MNCH sector of Tanzania. 2. no patients or community members were included in any of the data collection activities 3. cultural barriers and local contextual factors may have prevented a more robust understanding of the data | | |
| **If this study is being excluded give reason?** | Not excluded | | |
| **Conclusion** | The methods used to inform the implementation strategies discussed in this report and many of the barriers, facilitators, and resultant implementation strategies identified regarding WHO MNH guidelines are transferable to other priority areas and guidelines. Therefore, these findings can inform and be integrated into future barrier and facilitator assessments and guideline implementation planning initiatives in Tanzania. | | |
| **Recommendations /policy implications** | 1. Require that request and reporting of drugs is completed on time to minimize stock-outs. 2. Re-distribute health care workers internally (within institutions) and externally (across institutions) to concentrate on maternal and newborn health services. To do this, all health care workers should be sufficiently trained to work in different clinical areas when required. 3. Promote linkage of services between facilities. Women can be linked to clinical postnatal care by medical officer. 4. Provide guideline-relevant training (see below) to nursing and midwives, and adapt policies to empower them to approach physicians when a woman’s health is at risk and to be part of the decision making process. 5. Educate pregnant women on aspects of their care during labour to help increase patient knowledge. Use mass media to promote awareness of the benefits of the recommendations | | |

### **Table S8.19**

| **Name of Journal**  African Journal of Reproductive Health | **Date of review** | **Year of Publication**  Jun, 2014 | **Unique form ID:**  SR-019 |
| --- | --- | --- | --- |
| **Title of publication** | Community level distribution of misoprostol to prevent Postpartum hemorrhage at home births in Northern Nigeria | | |
| **Author(s)** | Clara Ejembi, Oladapo Shittu, Molly Moran, Faraouk Adiri, Olugbenga Oguntunde, Babalafia Saadatu, Idris Hadiza, Larai Aku-Akai, Victor Ajayi, Ndola Prata | | |
| **Study settings** | Country: Northern Nigeria  Rural/Urban: Rural  Health facility/ community based: community based  Public/Private facility: Nil | | |
| **Study Design** | Before and after study design using community based participatory approached | | |
| **Data collection Methodology and tool** | community dialogues, interviewer administered questionnaire | | |
| **Targeted Audience** | Health care administrators, policymakers, non-governmental organization staff, representatives from professional associations, frontline health care providers (e.g., physicians, nurses, and midwives), and researchers/academics. | | |
| **Variable/ domain of interest** | Uterotonic coverage at birth, feasibility of the intervention | | |
| **What implementation gaps or barriers were identified?** | 1. Cultural and structural barrier limits coverage. | | |
| **What are the advantages mentioned of implementing misoprostol for PPH or PAC?** | Heat stable, easy to administered | | |
| **What is/ are the of limitation/s of this study** | Not mentioned | | |
| **If this study is being excluded give reason?** | Not excluded | | |
| **Conclusion** | Introducing community drug keeper as an intervention that can provide life-saving uterotonic protection to women who deliver at home, thus increase the coverage. Key findings of this study can continue to inform policy makers in Nigeria and can be a part of evidence based on how to scale up this critical maternal health intervention throughout the country. | | |
| **Recommendations /policy implications** | 1. Need for a Policy change and future implementation of community level misoprostol distribution using the findings of this study to scale up this approach | | |

### **Table S8.20**

| **Name of Journal**  Bio Med Central | **Date of review** | **Year of Publication**  2016 | **Unique form ID:**  SR-020 |
| --- | --- | --- | --- |
| **Title of publication** | Navigating barriers: two-year follow up on recommendations to improve the use of maternal health guidelines in Kosovo | | |
| **Author(s)** | Julia E. Moore, Sami Uka, Joshua P. Vogel, Caitlyn Timmings, Shusmita Rashid, A. Metin Gülmezoglu and Sharon E. Straus | | |
| **Study settings** | Country: Kosovo  Rural/Urban: both  Health facility/ community based: Policy level  Public/Private facility: both | | |
| **Study Design** | Realistic Evaluation approach, using qualitative approach | | |
| **Data collection Methodology and tool** | FGD’s, IDI’s. | | |
| **Targeted Audience** | Local stakeholders (policy makers, researchers, academia, frontline health care providers, representative from professional associations and NGO’s representatives) | | |
| **Variable/ domain of interest** | Uterotonic coverage at birth, feasibility of the intervention | | |
| **What implementation gaps or barriers were identified?** | 1. Lack of guidelines and protocols, 2. There is lack of awareness and clarity regarding difference between protocol and guidelines. 3. Lack of decision making power among stake-holders to reach a final decision. 4. Lack of motivation among providers due to lack of clarity and no monetary incentives. 5. lack of communication between key stakeholder groups | | |
| **What are the advantages mentioned of implementing misoprostol for PPH or PAC?** | Safety and efficiency | | |
| **What is/ are the of limitation/s of this study** | 1. Time-limited grants, little research is done to evaluate the long-term activities and impacts of implementation efforts. 2. Communication challenges between groups were identified as a major barrier in 2012 and continue to pose challenges. 3. Finally, our understanding and interpretation of the data were limited by cultural barriers and local contextual factors | | |
| **If this study is being excluded give reason?** | Not excluded | | |
| **Conclusion** | Using a realist evaluation approach, This study assessed activities and progress over a two-year period on the nine recommendations designed to support Kosovo in implementing the WHO guideline on the prevention and treatment of PPH. The evaluation findings provided rich insights into the barriers and opportunities experienced to date.  Reporting on process evaluation findings, particularly in the context of implementation initiatives, is important for providing insight into realistic targets and results for changes in behavioral outcomes. It is beneficial to share these lessons learned throughout the implementation process for use in other settings. Next steps will involve evaluating how operationalizing the five recommendations has affected outcomes related to use of the WHO prevention and treatment of PPH guideline in Kosovo. Once data monitoring systems are established, future evaluations will examine the degree of WHO maternal health guideline implementation. | | |
| **Recommendations /policy implications** | 1. Establish a mechanism for continuous communication, collaboration, and experience-sharing between key stakeholder groups (e.g., maternal and child health forum); and 2. Foster teamwork culture and collective responsibility rather than focusing on individualization and personalization; this shift could focus on building motivation and using incentivized strategies, rather than focusing on punishment. | | |

## **Table S9:** **List of Excluded and Included Studies:**

| **Code. No** | **Title of the Study** | **Author/year** | **Status** | **Justification for inclusion or exclusion** |
| --- | --- | --- | --- | --- |
| SR-001 | Implementation of misoprostol for post abortion care in Kenya and Uganda: a qualitative evaluation. | Osur, J., Baird, T. L., Levandowski, B. A., Jackson, E., & Murokora, D. (2013). | Included | Implementation gaps specific to misoprostol were identified |
| SR-002 | Assessing post abortion care in health facilities in Afghanistan : a cross sectional study | Ansari, N., Zainullah, P., Kim, Y. M., Tappis, H., Kols, A., Currie, S., Stekelenburg, J. (2015) | Included | Implementation gaps specific to misoprostol were identified |
| SR-003 | Knowledge, attitudes and practices related to uterotonics drugs during childbirth in Karnataka, India: A Qualitative research study | Deepak, N. N., Mirzabagi, E., Koski, A., & Tripathi, V. (2013). | Included | Implementation gaps specific to misoprostol were identified |
| SR-004 | Helping rural women in Pakistan to prevent postpartum hemorrhage: A quasi experimental study | Mir, A. M., Wajid, A., & Gull, S. (2012). | Included | Gaps identified specific to misoprostol |
| SR-005 | Report on Management of Postpartum Hemorrhage - Findings from a survey with 69 FIGO Member Associations, 2016 | FIGO Member associations. | Included | Implementation gaps specific to misoprostol were identified |
| SR-006 | Modeling maternal mortality in Bangladesh: the role of misoprostol in postpartum hemorrhage prevention | Prata, N., Bell, S., & Quaiyum, M. A. (2014). | Included | Implementation gaps specific to misoprostol were identified |
| SR-007 | Misoprostol for postpartum hemorrhage: Moving from evidence to practice | Starrs, A., & Winikoff, B. (2012). | Included | Gaps specific to misoprostol identified |
| SR-008 | Is attendant at delivery associated with the use of interventions to prevent postpartum hemorrhage at home births? The case of Bangladesh | Prata, N., Bell, S., Holston, M., & Quaiyum, M. A. (2014) | Included | Implementation gaps specific to misoprostol were identified |
| SR-009 | Are national policies and programs for prevention and management of postpartum hemorrhage and preeclampsia adequate? A key informant survey in 37 countries | Smith, J. M., Currie, S., Cannon, T., Armbruster, D., & Perri, J. (2014). | Included | Implementation gaps specific to misoprostol were identified |
| SR-010 | Shaping legal abortion provision in Ghana: Using policy theory to understand provider-related obstacles to policy implementation | Aniteye, P., & Mayhew, S. H. (2013). | Included | Implementation gaps specific to misoprostol were identified |
| SR-011 | Global causes of maternal death: a WHO systematic analysis | Say, L., Chou, D., Gemmill, A., Tunçalp, Ö., Moller, A. B., Daniels, J., …& Alkema, L. (2014). | Excluded | No implementation gaps particular to misoprostol or uterotonics were identified in this study |
| SR-012 | Misoprostol for Prevention of Postpartum Hemorrhage at Home Birth in Afghanistan: Program Expansion Experience | Haver, J., Ansari, N., Zainullah, P., Kim, Y. M., & Tappis, H. (2016). | Included | Implementation gaps specific to misoprostol were identified |
| SR-013 | The UN Commission on Life Saving Commodities 3 years on: global progress update and results of a multi-country assessment. | Pronyk, P. M., Nemser, B., Maliqi, B., Springstubb, N., Sera, D., Karimov, R., & Leads, U. A. (2016). | Included | Although this analysis has a focused on 13 lifesaving commodities; misoprostol is one of them, this situation analysis has also identified gaps and barriers |
| SR-014 | Prevention of Postpartum Hemorrhage in rural Ethiopia | Report prepared by Technology Transfer and Research Translation Directorate at the Ethiopian Health and Research institute | Included | Implementation gaps specific to misoprostol were identified |
| SR-015 | Low-and middle-income countries face many common barriers to implementation of maternal health evidence products. | Ritchie, L. M. P., Khan, S., Moore, J. E., Timmings, C., van Lettow, M., Vogel, J. P. & Uka, S. (2016). | Included | Implementation gaps specific to misoprostol were identified |
| SR-016 | Interpretation of National Policy Regarding Community‐Based Use of Misoprostol for Postpartum Hemorrhage Prevention in Ethiopia: A Tale of Two Regions. | Spangler, S. A., Gobezayehu, A. G., Getachew, T., & Sibley, L. M. (2014). | Included | Implementation gaps specific to misoprostol were identified |
| SR-017 | Progress and gaps in reproductive health services in three humanitarian settings: mixed-methods case studies, | Casey, S.E., Chynoweth, S, K., Cornier, N., Gallagher, M.C., & Wheeler, E.E. (2015) | Included | Gaps specific to misoprostol identified |
| SR-018 | Understanding barriers and facilitators to implementation of Maternal health guidelines in Tanzania: A great network Research activity | Khan, S., Timmings, C., Vogel, J., Islam, S., Puchalski, L., & Straus, S. E. | Included | Gaps specific to misoprostol identified |
| SR-019 | Community level distribution of misoprostol to prevent Postpartum hemorrhage at home births in Northern Nigeria | Ejembi, C., Shittu, O., Moran, M., Adiri, F., Oguntunde, O., Saadatu, B., & Williams, N. (2014). | Included | Gaps specific to misoprostol identified |
| SR-020 | Navigating barriers: two-year follow up on recommendations to improve the use of maternal health guidelines in Kosovo | .Moore, J. E., Uka, S., Vogel, J. P., Timmings, C., Rashid, S., Gülmezoglu, A. M., & Straus, S.E. (2016). | Included | Gaps specific to misoprostol identified |

## **Table S10: Methodological details and key findings of the included studies:**

| **StudyCode** | **Citation** | **Objective of the study** | **Study Country/ setting** | **Study design and data collection method** | **Key findings/Implementation Barriers /gaps identified** |
| --- | --- | --- | --- | --- | --- |
| SR-001 | Osur, J., Baird, T. L., Levandowski, B. A., Jackson, E., & Murokora, D. (2013). Implementation of misoprostol for post abortion care in Kenya and Uganda: a qualitative evaluation. *Global Health Action*, *6*,10.3402/gha.v6i0.19649.<http://doi.org/10.3402/gha.v6i0.19649> | 1. To evaluate the effectiveness of the process used in introducing MPAC in the health system; 2. To approximate the minimum capacity (infrastructure, equipment, supplies, and human resources) required in health facilities to provide MPAC; 3. To assess patient satisfaction with MPAC from the perspectives of the care providers; | Kenya and Uganda (facility based) | Qualitative, utilizing In depth interviews | 1. Lack of national policies and guidelines for MPAC. 2. Registration of misoprostol specifically for PAC 3. Procuring misoprostol and maintain adequate supplies. 4. Inadequate staffing of facilities with providers trained in MPAC |
| SR-002 | Ansari, N., Zainullah, P., Kim, Y. M., Tappis, H., Kols, A., Currie, S., Stekelenburg, J. (2015). Assessing post-abortion care in health facilities in Afghanistan: a cross-sectional study. *BMC Pregnancy and Childbirth*, *15*, 6. <http://doi.org/10.1186/s12884-015-0439-x> | To assess the public health system’s readiness to provide PAC Services | Afghanistan  (facility based) | A cross-sectional study. Quantitative methodology, using Averting Maternal Death and Disability Program Needs Assessment Toolkit and National Midwifery education assessment tools | 1. Insufficient Supplies and equipment needed for PAC 2. Capacity of healthcare provider to deliver this service. 3. Insufficient knowledge and skills of PAC service providers 4. Limited coverage of PAC in pre-service medical and midwifery education program 5. Stigma associated with its abortion inducing properties 6. legal restrictions that only permits pregnancy termination to save life of mother 7. Preferring MVA over Medical Abortion (MA) using misoprostol. 8. Use for paracetamol for post-procedure pain instead of misoprostol |
| SR-003 | Deepak, N. N., Mirzabagi, E., Koski, A., & Tripathi, V. (2013). Knowledge, Attitudes, and Practices Related to Uterotonic Drugs during Childbirth in Karnataka, India: A Qualitative Research Study. *PloS ONE*, *8*(4), e62801. <http://doi.org/10.1371/journal.pone.0062801> | To address gaps in term of health care providers and community members’ knowledge, attitudes and practices regarding uterotonics use during labor and delivery from the community to the facility level in Karnataka. | India  (community and health facility based) | Exploratory study,  Qualitative method using semi-structure in-depth interviews | 1. Misconception and knowledge gap of informal providers and community members (they belief increase pain is needed to facilitate delivery which is achieved by using oxytocin injection). 2. Conflicting perspective on uterotonic demand. 3. Inconsistency of provider knowledge related to its dosage, required monitoring, and its administration.   . |
| SR-004 | Mir, A. M., Wajid, A., & Gull, S. (2012). Helping rural women in Pakistan toprevent postpartum hemorrhage: A quasi experimental study.*BMC Pregnancy and Childbirth*, *12*, 120.<http://doi.org/10.1186/1471-2393-12-120> | To document feasibility of distribution of misoprostol by community based provider. To assess the acceptability and use of misoprostol by pregnant women for prevention of PPH | Pakistan, rural area (home based settings) | Quasi Experimental study design comprising intervention and comparison area | 1. Hindrance from relatives in taking misoprostol (opposing view) 2. Lack of knowledge of study participants regarding timings of taking misoprostol and appropriate timings for referrals |
| SR-005 | Report on Management of Postpartum Hemorrhage - Findings from a survey with 69 FIGO Member Associations, 2016 | To find out about countries’ national guidelines for PPH, and inclusion of key PPH medicines on national EMLs as well as any challenges to implementing evidence-based practice in order to further support national FIGO Member Associations in their work towards their maternal health goals | Developing countries (69 FIGO Countries) | Web based survey using email | 1. Healthcare providers don’t know about guidelines. 2. Misoprostol not included in National EML. 3. No guideline for misoprostol specific for PPH. 4. No registration of misoprostol specific for PPH |
| SR-006 | Prata, N., Bell, S., & Quaiyum, M. A. (2014). Modeling maternal mortality in Bangladesh: the role of misoprostol in postpartum hemorrhage prevention.*BMC Pregnancy and Childbirth*, *14*, 78. <http://doi.org/10.1186/1471-2393-14-78> | 1. To examine potential role misoprostol can play in decline of maternal deaths attributed to PPH in Bangladesh. 2. To evaluate the feasibility and acceptability of scaling up community based use of misoprostol. | Bangladesh (home births/ community based) | Operational research , Using  Qualitative verbal autopsy questionnaire | Little knowledge of community regarding causes of PPH and using misoprostol in such conditions. |
| SR-007 | Starrs, A., & Winikoff, B. (2012). Misoprostol for postpartum hemorrhage: Moving from evidence to practice. International Journal of Gynecology & Obstetrics, 116(1), 1-3. | To assess and address coverage gaps in using misoprostol | Low resource regions | Special communication | 1. Association with abortion endanger discomfort and resistance among policy makers and providers. 2. No registration of misoprostol for other than management of gastric ulcer. 3. Lack of clear and consistent usage guidance often outdated, non-evidence based protocol in practice. 4. Fear of its promotion, it may deter women from seeking care at facilities with skilled providers. 5. It is perceived that if misoprostol is widely available, it will be repurposed for used in medical abortion, thus increasing number of abortions. |
| SR-008 | Prata, N., Bell, S., Holston, M., & Quaiyum, M. A. (2014). Is attendant at delivery associated with the use of interventions to prevent postpartum hemorrhage at home births? The case of Bangladesh. *BMC Pregnancy and Childbirth*, *14*, 24. <http://doi.org/10.1186/1471-2393-14-24> | To determine whether the attendant at home delivery (TBA) trained on PPH interventions, TBA not trained on interventions, or lay attendant, which includes a family member or (neighbor) is associated with the use of interventions to prevent PPH at birth | Bangladesh (community based) | Cross-sectional study  Quantitatively, using Ante-natal care (ANC) cards | Inadequate Coverage of SBAs and TBA’s |
| SR-009 | Smith, J. M., Currie, S., Cannon, T., Armbruster, D., & Perri, J. (2014). Are national policies and programs for prevention and management of postpartum hemorrhage and preeclampsia adequate? A key informant survey in 37 countries. *Global Health, Science and Practice*, *2*(3), 275–284. <http://doi.org/10.9745/GHSP-D-14-00034> | To provide a global snapshot of the extent to which the essential policies and programs were in place and to provide program managers and development partners with evidence on the key processes that facilitate scale-up and expansion of maternal health interventions, especially evidence based PPH | 37 Developing countries  (Policy/ National level) | Cross-sectional  mixed method using Key informants survey and questionnaire | 1. More countries included oxytocin in essential medicine list than misoprostol. 2. Inconsistent and limited availability of misoprostol limits implementation of national priorities. 3. Misoprostol use to prevent PPH at home birth have piloted in some countries but far fewer have taken the strategy to scale up. 4. Barriers to access and availability. 5. Paying for medicine is a bottle neck to improve coverage despite to be inexpensive. 6. Technical inconsistencies in national service delivery guidelines (SDG) – incomplete and out dated. 7. Lack of national reporting on HMIS on use of uterotonics. 8. Limited scope of practice of midwifes 9. Gaps in inclusion of maternal health indicators in national data. |
| SR-010 | Aniteye, P., & Mayhew, S. H. (2013). Shaping legal abortion provision in Ghana: using policy theory to understand provider-related obstacles to policy implementation. *Health Research Policy and Systems*, *11*, 23. <http://doi.org/10.1186/1478-4505-11-23>. | To investigate the reasons for poor implementation of the policy in Ghana using Lipsky’s theory of street level bureaucracy to better understand how providers shape and implement policy and how providers-level barriers might be overcome | Ghana  (Policy Level) | Qualitative , using In depth interview and document analysis | 1. Doubt about standard and protocols for abortion care. 2. Perceived lack of administrative support. 3. Judgmental attitudes because of personal, social and structural reasons |
| SR-012 | Haver, J., Ansari, N., Zainullah, P., Kim, Y. M., & Tappis, H. (2016). Misoprostol for Prevention of Postpartum Hemorrhage at Home Birth in Afghanistan: Program Expansion Experience. Journal of Midwifery & Women’s Health. | To determine the effectiveness of advance distribution of misoprostol for self-administration across 20 districts in Afghanistan and identify adverse events during expansion | Afghanistan  (community based) | Before and after design without control group  Baseline and End-line surveys, verbal autopsy questionnaire | 1. Lack of integration of advance distribution of misoprostol with Basic package of health services. 2. Scarcity of CHW’s or alternative cadre to reach women without access to existing health services. 3. Disparities in service utilization between rural and urban |
| SR-013 | Pronyk, P. M., Nemser, B., Maliqi, B., Springstubb, N., Sera, D., Karimov, R., & Leads, U. A. (2016). The UN Commission on Life Saving Commodities 3 years on: global progress update and results of a multicountry assessment. *The Lancet Global Health*, *4*(4), e276- | To review the progress against 3 main strategies ‘in 3 years   1. RMNCH Situational analysis. 2. commodity related and system related bottle-necks, 3. a country engagement process to provide technical and financial support to national RMNCH Plans | 12 sub- Saharan African countries  (National/Policy level) | Situation Analysis,  Review of key documents and reference data, interview with key stakeholders | 1. Commodity security strategies were often poorly developed. 2. Capacity to monitor the quality and safety of medicines is scarce. 3. Gaps in monitoring the distribution of commodities from national warehouse to service delivery site remain a barrier. 4. Fragmented supply chains. |
| SR-014 | Report on Prevention of Postpartum Hemorrhage in rural Ethiopia, March, 2012 | To inform policy makers and stakeholders to systematically and transparently consider the the best available evidence regarding prevention of postpartum hemorrhage in rural settings and to engage them in deliberations about those policies | Ethiopia | Situational analysis using document review | 1. Misuse of misoprostol outside the intended purpose for labor induction. 2. Inadequate logistics due to poor road infrastructure, and poor setting for procurement in remote areas. 3. Financial constraints in term of training TBA’s, cost of drug. 4. Shortage of TBA’s |
| SR-015 | Ritchie, L. M. P., Khan, S., Moore, J. E., Timmings, C., van Lettow, M., Vogel, J. P. & Uka, S. (2016). Low-and middle-income countries face many common barriers to implementation of maternal health evidence products. *Journal of clinical epidemiology*. | To explore similarities and differences in challenges to maternal health and evidence implementation in general across several low-and middle income countries (LMIC’s) and to identify common and unique themes representing barriers to and facilitators of evidence implementation in KMIC health care settings. | Kosovo, Myanmar, Malawi, Tanzania and Uganda  At all 3 levels) | analysis of qualitative data using  Meeting reports and articles describing projects undertaken in 5 LMIC’s from GREAT Network projects | Barriers at Health System level (related to PPH):   1. Lack of supplies especially in small/ rural centres. 2. Lack of ability to smoothly transfer patients/or coordinate care across health system levels. 3. Lack of ability to implement and monitor implementation and current practices.   Barriers at Provider Level:   1. Lack of training capacity and time to attend training. 2. Lack of awareness of the guidelines/evidence. 3. Lack of communication/inter-professional collaboration   Barriers at Community level:   1. Cultural practices and lack of health seeking behavior. 2. Ethnic and cultural differences 3. Patients’ lack of trust of lower-cadre health workers |
| SR-016 | Spangler, S. A., Gobezayehu, A. G., Getachew, T., & Sibley, L. M. (2014). Interpretation of National Policy Regarding Community‐Based Use of Misoprostol for Postpartum Hemorrhage Prevention in Ethiopia: A Tale of Two Regions. Journal of Midwifery & Women’s Health, 59(s1), S83-S90. | To examine understanding of national policy for community based use of misoprostol to prevent PPH in 2 regions of Ethopia | Ethopia  (Community based) | Qualitative study  In-depth interview and survey | 1. No policy specifies whether community health development agents, TBA’s, or laypersons can handle and administer misoprostol. 2. Confusion regarding the existence of official policy for community based use of misoprostol to prevent PPH. 3. Systematic inconsistencies in drug supply, training and professional (or personal) commitment among lower-level officials and health workers. 4. Confusion about the existence of specific policy for PPH prevention. 5. Fear or safety concerns includes administration in the event of an undiagnosed twin (leading to fatal outcome), administration during labour (cause uterine rupture), and use of unsafe abortion. 6. In response to advance distribution; fears concerning intra-partum administration, undiagnosed multiple gestation, unsafe abortion, medication sharing and possibility of its use after expiry. 7. It is believed that misoprostol will increase the home based delivery and limit the facility based delivery. 8. Lack of communication of policy that exist. |
| SR-017 | Casey, S.E., Chynoweth, S, K., Cornier, N., Gallagher, M.C., & Wheeler, E.E. (2015) Progress and gaps in reproductive health services in three humanitarian settings: mixed-methods case studies, Conflict & Health, 9(1), 1. | To document the current availability, quality, utilization of, and access barriers to RH services in selected humanitarian settings in order to contribute to the evidence base informing humanitarian health-related policy and programming. | Sub-Saharan Africa (Democratic Republic of Congo, Maban & Burkina Faso | Cross-sectional, mixed methods case study Quantitatively: Facility assessment checklist, clinical register review, room by room inventory check. Qualitatively: Focus group discussions (FGD’s) with community members | 1. Dearth of trained staff in providing PAC service 2. Manual vacuum aspiration is the most commonest mean for uterine evacuation in Burkina Faso and DRC 3. FGDs revealed negative attitudes toward abortion, which they said conflicted with religious beliefs. 4. Availability of PAC and a full package of FP services were limited in health centers. 5. paucity of drugs as the primary barrier to providing adequate RH care 6. lack of non-technical skills such as poor situation awareness, decision-making, and inter-personal skills including communication and teamwork |
| SR-018 | Khan, S., Timmings, C., Vogel, J., Islam, S., Puchalski,L., & Straus, S. E. Understanding Barriers and Facilitators to Implementation of Maternal Health Guidelines in Tanzania: A Great Network Research Activity. | 1.Providing key recommendations to inform the development of a multi-level implementation strategy for improving use of guidelines nationally;  2. Supporting local stakeholders in the development and delivery of the implementation strategy; 3. Supporting local stakeholders in the development of a monitoring and evaluation plan to assess impact of guideline implementation | Tanzania | Mixed Method study design using FGD and ranking exercise | *Barriers at Health system level*   1. Limited access to drugs and challenges in the drug distribution system were identified as barriers to the successful implementation. 2. A shortage of health care providers was described as a barrier experienced throughout the health care system in Tanzania 3. Lack of ability to smoothly transfer patients/or coordinate care across health system levels. 4. Lack of awareness of the existence of guidelines by health care workers.   *Barriers at Provider Level:*   1. fear that misoprostol can be misused for abortion: 2. inadequacies in the training curriculum may contribute to lack of compliance with guideline   Barriers at Community level:   1. Limited health-seeking behavior and patient preference for care |
| SR-019 | Ejembi, C., Shittu, O., Moran, M., Adiri, F., Oguntunde, O., Saadatu, B., & Williams, N. (2014). Community-level distribution of misoprostol to prevent postpartum hemorrhage at home births in northern Nigeria. African journal of reproductive health, 18(2), 166-175. | 1. To determine effective distribution outlets and persons to administer misoprostol to parturient women at community level. 2. To determine the acceptability and uptake of misoprostol to prevent PPH. 3. To explore issues related to sustainability of community level distribution of misoprostol | Northern Nigeria | Before and after study design using community based participatory approached using community dialogues | Cultural and structural barrier limits coverage |
| SR-020 | Moore, J. E., Uka, S., Vogel, J. P., Timmings, C., Rashid, S., Gülmezoglu, A. M., & Straus, S. E. (2016). Navigating barriers: two-year follow up on recommendations to improve the use of maternal health guidelines in Kosovo. BMC Public Health, 16(1), 987. | The aim of the current study was to conduct a qualitative process evaluation, using a realist evaluation approach of activities and progress on the nine recommendations made to prepare Kosovo to implement the WHO prevention and treatment of PPH guideline | Kosovo | Realistic Evaluation approach, using qualitative approach using FGD’s and IDI’s | 1. Lack of guidelines and protocols, 2. There is lack of awareness and clarity regarding difference between protocol and guidelines. 3. Lack of decision making power among stake-holders to reach a final decision. 4. Lack of motivation among providers due to lack of clarity and no monetary incentives. 5. lack of communication between key stakeholder groups 6. a focus on punitive interventions (rather than taking a more positive, motivational approach) |

## **Table S11: AMSTAR- a measurement tool to assess the methodological quality of Systematic Review**

| **S. No** | **AMSTAR Criteria** | **Status**  **(Yes, No, Can’t answer, Not Applicable)** | **Score** |
| --- | --- | --- | --- |
| 1 | Was an “a priori” design provided? | Yes | 1 |
| 2 | Was there duplicate study selection and data extraction? | Not Applicable | N/A |
| 3 | Was a comprehensive literature search perform? | Yes | 1 |
| 4 | Was the status of publication (i.e. grey literature) used as an inclusion criteria? | Yes | 1 |
| 5 | Was the list of studies included and excluded provided? | Yes | 1 |
| 6 | Were the characteristics of the included studies provided? | Yes | 1 |
| 7 | Was the scientific quality of the included studies assessed and documented? | Yes | 1 |
| 8 | Was the scientific quality of the included studies used appropriately in formulating conclusions? | Yes | 1 |
| 9 | Were the methods used to combine the findings of studies appropriate? | Yes | 1 |
| 10 | Was the likelihood of publication biased assessed | Not applicable | N/A |
| 11 | Was the conflict of interest included | Yes | 1 |
| Total Score | | | 9 |
