# Supplementary figures and images for: Barriers or gaps in implementation of misoprostol use for post-abortion care and post-partum hemorrhage prevention in developing countries: a systematic review
Source: Reprod Health. 2017 Oct 27;14:139. doi: 10.1186/s12978-017-0383-5 (PMC5659002; doi:10.1186/s12978-017-0383-5)

**ERC Approval/Exemption Letter**


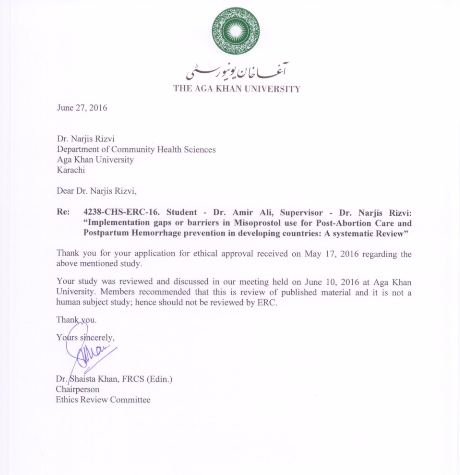


**Waiver Letter**

Supplement: Supplementary file 3 — ERC Approval / exemption letter. (DOCX 771 kb) [file 12978_2017_383_MOESM3_ESM.docx]
